# Genomic copy number variation association study in Caucasian patients with nonsyndromic cryptorchidism

**DOI:** 10.1186/s12894-016-0180-4

**Published:** 2016-10-21

**Authors:** Yanping Wang, Jin Li, Thomas F. Kolon, Alicia Olivant Fisher, T. Ernesto Figueroa, Ahmad H. BaniHani, Jennifer A. Hagerty, Ricardo Gonzalez, Paul H. Noh, Rosetta M. Chiavacci, Kisha R. Harden, Debra J. Abrams, Deborah Stabley, Cecilia E. Kim, Katia Sol-Church, Hakon Hakonarson, Marcella Devoto, Julia Spencer Barthold

**Affiliations:** 1 Nemours Biomedical Research, Nemours /Alfred I. duPont Hospital for Children, Wilmington, DE 19803 USA; 2 Division of Urology, Nemours/Alfred I. duPont Hospital for Children, Wilmington, DE 19803 USA; 3 Center for Applied Genomics, The Children’s Hospital of Philadelphia, Philadelphia, PA 19104 USA; 4 Division of Urology, The Children’s Hospital of Philadelphia, Philadelphia, PA 19104 USA; 5 Division of Genetics, The Children’s Hospital of Philadelphia, Philadelphia, PA 19104 USA; 6 Department of Pediatrics, Perelman School of Medicine, University of Pennsylvania, Philadelphia, PA 19104 USA; 7 Department of Biostatistics and Epidemiology, Perelman School of Medicine, University of Pennsylvania, Philadelphia, PA 19104 USA; 8 Department of Molecular Medicine, Sapienza University, Rome, Italy; 9 Present address: Auf der Bult Kinder- und Jugendkrankenhaus, Hannover, Germany; 10 Present address: Division of Pediatric Urology, Cincinnati Children’s Hospital Medical Center, Cincinnati, OH USA

**Keywords:** Cryptorchidism, Genetics, CNV

## Abstract

**Background:**

Copy number variation (CNV) is a potential contributing factor to many genetic diseases. Here we investigated the potential association of CNV with nonsyndromic cryptorchidism, the most common male congenital genitourinary defect, in a Caucasian population.

**Methods:**

Genome wide genotyping were performed in 559 cases and 1772 controls (Group 1) using Illumina HumanHap550 v1, HumanHap550 v3 or Human610-Quad platforms and in 353 cases and 1149 controls (Group 2) using the Illumina Human OmniExpress 12v1 or Human OmniExpress 12v1-1. Signal intensity data including log R ratio (LRR) and B allele frequency (BAF) for each single nucleotide polymorphism (SNP) were used for CNV detection using PennCNV software. After sample quality control, gene- and CNV-based association tests were performed using cleaned data from Group 1 (493 cases and 1586 controls) and Group 2 (307 cases and 1102 controls) using ParseCNV software. Meta-analysis was performed using gene-based test results as input to identify significant genes, and CNVs in or around significant genes were identified in CNV-based association test results. Called CNVs passing quality control and signal intensity visualization examination were considered for validation using TaqMan CNV assays and QuantStudio® 3D Digital PCR System.

**Results:**

The meta-analysis identified 373 genome wide significant (*p* < 5X10^−4^) genes/loci including 49 genes/loci with deletions and 324 with duplications. Among them, 17 genes with deletion and 1 gene with duplication were identified in CNV-based association results in both Group 1 and Group 2. Only 2 genes (*NUCB2* and *UPF2*) containing deletions passed CNV quality control in both groups and signal intensity visualization examination, but laboratory validation failed to verify these deletions.

**Conclusions:**

Our data do not support that structural variation is a major cause of nonsyndromic cryptorchidism.

**Electronic supplementary material:**

The online version of this article (doi:10.1186/s12894-016-0180-4) contains supplementary material, which is available to authorized users.

## Background

Nonsyndromic cryptorchidism, or isolated undescended testis, is one of the most common pediatric congenital anomalies, affecting 2-3 % of boys, and is associated with infertility and testicular malignancy later in life [1]. The etiology is largely unknown and likely multifactorial. Familial clustering suggests moderate genetic contribution to the disease [2].

A candidate approach to gene discovery has revealed some potential risk genes, but the results are inconsistent and population-specific [3–10]. Recently we performed a genome-wide association study (GWAS) in 912 nonsyndromic cryptorchidism cases and 2921 controls [11, 12] to identify common allelic variants across the genome associated with the disease. No variant reached genome-wide significance (*p* ≤ 7X10^−9^) in full analysis, and one variant (rs55867206, near *SH3PXD2B, p* = 2X10^−9^) passed this threshold in a subgroup analysis of proximal testis position. Pathway analysis of suggestive association markers (*p* ≤ 10^−3^) using several bioinformatics tools identified overrepresentation of genes/functions linked to cytoskeleton-dependent processes, syndromic cryptorchidism and hypogonadotropic hypogonadism.

Over the past decade, evidence has shown that copy number variation (CNV) plays an important role in the occurrence of many diseases [13]. Analysis of CNVs using array comparative genomic hybridization found *VAMP7* duplication and *OTX1* deletion in individuals with congenital genitourinary defects [14, 15], with cryptorchidism as one of the primary traits. However, the association of CNVs with nonsyndromic cryptorchidism has not been explored. Through analysis of GWAS data [11, 12], we hypothesized that CNV is a significant cause of nonsyndromic cryptorchidism in Caucasian males.

## Methods

### Subjects and genotyping

Cases were self-reported Caucasian subjects with nonsyndromic cryptorchidism who underwent surgical repair at Nemours/Alfred I. DuPont Hospital for Children (Nemours) or The Children’s Hospital of Philadelphia (CHOP). Subjects with multiple congenital anomalies or diagnosis of any syndrome, other genital anomalies (hypospadias, chordee or other penile anomalies) or abdominal wall defects were excluded from the study. Control subjects were recruited through the CHOP Health Care Network. They were self-reported Caucasian males who were at least 6 years old with no known history of testicular disease, penile anomaly, diagnosis of a syndrome or any additional medical disorder associated with cryptorchidism. Basic demographic and phenotypic data collected include age of diagnosis, race, ethnicity, laterality and the position of affected testes.

As described in detail in previous publications [11, 12], two groups of cases were genotyped at the Center for Applied Genomics at CHOP to match available control genotype data. In Group 1, 559 cases and 1772 controls were genotyped using the Illumina HumanHap550 v1, HumanHap550 v3 or Human610-Quad platforms that share over 535 K single nucleotide polymorphisms (SNPs) in common. In Group 2, 353 cases and 1149 controls were genotyped using the Illumina Human OmniExpress 12v1 or Human OmniExpress 12v1-1 platforms that share over 719 K SNPs. The global SNP and gene coverage of our SNP arrays are approximately 85 % and 80 %, respectively [16], and the average distance between probes is 4 kbp-5.5 kbp. At SNP genotype calling, cluster files (.egt) provided by Illumina were used as a common reference.

### CNV detection and sample quality control

Due to differences in SNP coverage and less than 310 K intersection of SNPs between platforms used in the 2 case–control groups, CNV detection, sample quality control (QC), and association tests were performed separately in Groups 1 and 2. We used the PennCNV software package [17–20] to make CNV calls based on signal intensity data from genotyping arrays including log R ratio (LRR) and B allele frequency (BAF) for each SNP. Adjacent CNV calls were then automatically examined and merged using PennCNV software.

We used sample QC criteria from our prior genome-wide genotyping data analysis in PLINK [11, 12, 21–23]. Individuals were excluded from further analysis if one of below criteria were met: (1) discordance between reported sex and sex chromosome SNP data; (2) missing genotype rate >3 %; (3) potential duplicates or relatives (based on estimate of proportion of alleles shared identical by descent >0.1875); and (4) non-Caucasian ancestry based on multidimensional scaling (MDS) analysis using data from the Stanford Human Genome Diversity Project (HGDP) [24, 25]. We removed all samples that deviated from the means of the first or second MDS components by more than 3 standard deviations (SD). We also used a sample quality control function implemented in the ParseCNV software package [26, 27] and removed samples with (1) high intensity noise (measured by SDLRR (SD of LRR) > mean +3 SD); (2) extreme intensity waviness (measured by more than 3 SD of mean of GCWF (Guanine-Cytosine base pair wave factor)) and (3) high number of CNV counts per sample (measured by CNV count number > mean + 3 SD).

### Gene based association analysis, meta-analysis and CNV based association analyses

Given that SNP overlap is low between the genotyping platforms used in Group 1 and 2, and the uncertainty of CNV boundary data from different platforms, we were unable to directly merge CNV from the two groups. Therefore, after removing individuals not passing samples QC, we performed gene-based association tests separately in Group 1 and 2 samples using the ParseCNV software package. We then performed meta-analyses of gene-based association results with METAL software [28, 29] using gene names as markers to identify significant genes (*p* < 5X10^−4^, a conservative bar for CNV genome-wide significance suggested by ParseCNV). We also performed CNV-based association tests in cleaned Group 1 and 2 samples using ParseCNV software package. CNVs in or around significant genes from the gene-based meta-analyses were identified by searching the “gene” column in CNV-based association tests results. The CNVs were considered not passing CNV QC and removed if one of below criteria were met: average number of probes in CNV (AvgProbes) < 5, worst p-value in the span of CNV calls contributing to the significant CNV region (PenMaxP) > 0.5 and high frequency (Freq >0.5), nearly identical segmental duplications (SegDups) > 10, any locus frequently found in multiple studies such as T-cell receptor gene, human major histocompatibility complex gene etc. (Recurrent), the same inflated sample driving multiple CNV association signals (FreqInflated), the HMM confidence score in PennCNV calling (AvgConf) < 10, and allele A or B banding (ABFreq) in BAF low for duplications. Additionally, if more than three of below criteria were met, the CNV also was not considered for further analysis: CNV residing at centromere or telomere regions (TeloCentro), high or low GC content regions (AvgGC <30 or >60), CNV regions with high population frequency (PopFreq) >0.01, a large gap in probe coverage exists within CNV association signals (Sparse) >50 kbp, and average length of CNV <10 kbp [27].

### CNV visualization, examination and laboratory validation

CNVs passing QC in both Groups 1 and 2 were examined by the plots of signal intensity (LRR/BAF) generated using the CNV visualization function implemented in the PennCNV package. Three CNVs passed CNV quality control in both groups and signal intensity visualization examination, and were chosen for further validation using TaqMan CNV probes located in the central region of each CNV (Hs04383175_cn, Hs06286795_cn and Hs06269635_cn), TaqMan CNV reference assay (human RNase P: 4403326) and QuantStudio® 3D Digital PCR System (Thermo Fisher Scientific, Waltham, MA USA) by the Nemours Biomolecular Core Laboratory, following the manufacturer’s standard protocol.

## Results

Based on sample quality control criteria, 66 cases and 186 controls were removed, leaving 493 cases and 1586 controls in Group 1. In Group 2, 46 cases and 47 controls were removed, leaving 307 cases and 1102 controls. In Group 1, 7,376 deletions and 4,313 duplications were detected and 6,689 deletions and 6,635 duplications were detected in Group 2.

**Fig. 1:**
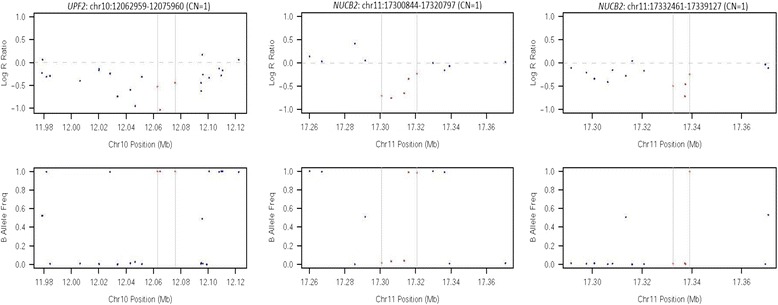
Array plot of Log R ratio and B allele frequency for *NUCB2* and *UPF2*

In gene-based association tests, 25 and 106 genes/loci with deletion, and 371 and 177 genes/loci with duplication reached genome-wide significance (*p* < 5x10^−4^) in Group 1 and Group 2 (Additional file 1). After meta-analysis, 49 genes/loci with deletion and 331 genes/loci with duplication reached genome-wide significance (Additional file 2). For 49 genes with deletion, the direction of effect was consistent in the two groups. The direction of effect was inconsistent for 6 duplications and no direction was given in one duplication which was due to *p* = 1 for that gene in gene-based association test of Group 2, and they were removed from further consideration, leaving 324 genes/loci with duplication. Among these 373 significant genes/loci, 17 with deletion and 1 with duplication were identified in CNV-based association analysis in both Group 1 and Group 2 (Table 1). Five genes/loci (TCR gamma alternate reading frame protein (*TARP*), tonsoku-like DNA repair protein (*TONSL*), TONSL antisense RNA 1 (*TONSL-AS1*), nucleobindin 2 (*NUCB2*), and UPF2 regulator of nonsense transcripts homolog (yeast) (*UPF2*)) with deletion passed CNV quality control in both groups (Table 1). Signal intensity plots of CNVs in *NUCB2* and *UPF2* (Fig. 1: Array plot of Log R ratio and B allele frequency for *NUCB2* and *UPF2*) suggested heterozygous deletions: the LRR decrease below 0 and the BAF cluster around either 0 or 1, but not near 0.5. Signal intensity plots of CNVs in *TARP* and *TONSL*/*TONSL-AS1* did not pass visualization examination (Additional file 3: Array plot of Log R ratio and B allele frequency for *TARP* and *TONSL*/*TONSL-AS1*) due to LRR close to 0, BAF cluster near 0.5, or both. Thus only CNVs in *NUCB2* and *UPF2* were further considered in our study.

**Table 1:** Genes significant in meta-analysis and identified in CNV-based association tests

Gene Name	CNV Type	Group 1						Group 2					
		CNVR(hg19)	TagSNP	*P* value	Cases #	Control #	CNV QC Pass/Fail	CNVR(hg19)	TagSNP	*P* value	Cases #	Control #	CNV QC Pass/Fail
BBS5, KLHL41	Deletion	chr2:170354790–170368798	rs3769772	1.86E-11	30	11	PASS	chr2:170343083–170840224	rs2592804	0.217885025	1	0	FAIL
CDK19	Deletion	chr6:110972494–111696091	rs12198236	0.01327263	3	0	FAIL	chr6:111061814–111381468	rs9374202	0.010264833	3	0	PASS
SYNE1	Deletion	chr6:152938025–152969462	rs6940651	0.237247353	1	0	FAIL	chr6:152511420–152516441	rs7772542	2.04E-09	13	0	FAIL
TARP^a^	Deletion	chr7:38357194–38364605	rs11765884	3.74E-05	18	13	PASS	chr7:38341226–38341925	rs2736973	2.56E-07	29	26	PASS
TONSL-AS1, TONSL^a^	Deletion	chr8:144992103–146235564	rs11136344	0.027970085	7	7	PASS	chr8:145660543–145666578	rs2306384	5.04E-05	12	6	PASS
UPF2^a^	Deletion	chr10:12028228–12076043	rs7899260	0.00313275	4	0	PASS	chr10:12062959–12075960	rs7072007	8.15E-06	9	1	PASS
TET1	Deletion	chr10:70342775–70775081	rs7071780	0.05619918	2	0	FAIL	chr10:70410630–70432644	rs10762236	9.01E-11	15	0	PASS
MICU1	Deletion	chr10:74250792–74534174	rs7912170	0.237133237	1	0	FAIL	chr10:74304790–74433626	rs7909573	4.56E-08	11	0	PASS
ADK	Deletion	chr10:76092179–76099557	rs10824151	5.31E-06	14	5	FAIL	chr10:76372037–77001459	rs4746209	0.047420164	2	0	FAIL
NUCB2^a^	Deletion	chr11:17300844–17320797	rs12419530	4.08E-05	7	0	PASS	chr11:17332461–17339127	rs10466382	0.034028702	3	1	PASS
LARP4	Deletion	chr12:49846626–51537196	rs10876136	0.237133237	1	0	FAIL	chr12:50768339–50807570	rs7296212	2.21E-05	7	0	PASS
AQR	Deletion	chr15:34261920–35227613	rs16954263	0.237133237	1	0	FAIL	chr15:35233730–35252767	rs4513050	2.33E-09	17	3	FAIL
PIGL	Deletion	chr17:15043999–16221319	rs7210224	0.237133237	1	0	FAIL	chr17:16196056–16207526	rs11868284	2.21E-05	7	0	PASS
PAFAH1B1	Deletion	chr17:1811983–2578648	rs9674799	0.056145117	2	0	FAIL	chr17:2534710–2561169	rs11078288	0.000478637	5	0	PASS
SLC19A1	Deletion	chr21:46298869–47863025	rs2330183	0.138704428	2	1	FAIL	chr21:46945024–46953292	rs4819128	9.13E-06	10	2	PASS
FOLH1B	Duplication	chr11:89123768–89405190	rs7929532	0.237133237	1	0	FAIL	chr11:89374850–89405190	rs7112871	3.71E-35	54	2	FAIL

^a^Genes/loci passed CNV QC in both groups

The CNVs detected in *NUCB2* are around 20 kbp and 6.7 kbp in Group 1 and Group 2, and they do not overlap. The Database of Genomic Variants (DGV) in The Hospital for Sick Children, a teaching hospital affiliated with the University of Toronto [30, 31] reported a 15 kbp deletion in 1 of 2026 individuals and a 719 bp deletion in 2 of 2504 individuals at the CNV region of Group 1 (chr11:17300844–17320797), and a 5 kbp deletion in 1 of 17421 individuals at the CNV region of Group 2 (chr11:17332461–17339127). Seven cases in Group 1 and 3 cases in Group 2 contained *NUCB2* deletions based on this analysis. The CNVs detected in *UPF2* were approximately 47.8 kbp and 13 kbp in Groups 1 and 2, respectively, and the 13 kbp segment is inside the 47.8 kbp segment. DGV reported a 47.8 kbp deletion in 2 of 17421 individuals in this CNV region detected in Group 1. Four cases in Group 1 and 9 cases in Group 2 contained *UPF2* deletions. The CNV confidence scores of *NUCB2* and *UPF2* for each case generated during CNV calling by PennCNV are shown in Table 2. The score range was 12 to 55, which is considered borderline reliable for CNV detection.

**Table 2:** Relevant validation information for genes (*UPF2* and *NUCB2*) passed CNV QC and signal intensity examination

Group	Gene Name	CNV (hg19)	Probe # in overlapping CNV region	CNV size (bp)	TaqMan CNV assays and location	Cases with deletion (CNV confidence score)
Group 1	*UPF2*	chr10:12028228–12076043	10	47815	Hs04383175_cn; Chr10:12063665	D10 (26)^a^, D29 (55)^a^, D34 (46), 1495 (27)
	*NUCB2*	chr11:17300844–17320797	5	19953	Hs06286795_cn; Chr11:17306162	D8 (24), D10 (28)^a^, D24 (24), D29 (30)^a^, D33 (26), D132 (27), D139 (30)
Group 2	*UPF2*	chr10:12062959–12075960	3	13001	Same as *UPF2* assay in group 1	7279 (15), 7334 (12)^a^, 7338 (23), 7339 (16), 7341 (14), 7370 (26)^a^, 7453 (16), 7475 (24), 7479 (17)
	*NUCB2*	chr11:17332461–17339127	4	6666	Hs06269635_cn; Chr11:17336218	7334 (17)^a^, 7361 (14), 7370 (12)^a^

Case IDs with underline: samples tested by TaqMan CNV assays for validation

^a^samples with called deletions in both genes (*UPF2* and *NUCB2*)

We attempted to validate CNVs in *NUCB2* and *UPF2* in affected cases using TaqMan CNV assays and QuantStudio® 3D Digital PCR System (Table 2). After validating the TaqMan CNV assays using 2 control DNAs without called CNVs in these regions, we tested 12 samples from Group 1 or 2 with called CNVs within these genes of interest (Table 2, noted in case IDs with underline). All 12 tested samples were diploid (Additional file 4), indicating that bioinformatically-called deletions were not validated by TaqMan CNV assays.

## Discussion

Cryptorchidism is the most common male congenital genitourinary defect. While it is a manifestation of many congenital defect syndromes [32–34], the majority of cases are nonsyndromic and of unclear etiology. Our previous genome-wide association analyses of SNP data suggest that cryptorchidism is associated with significant genetic heterogeneity [11, 12]. In the present study, we performed genome-wide CNV association analysis to identify the potential association of structural variation with the occurrence of nonsyndromic cryptorchidism, and our results suggest that CNVs do not contribute to the genetic basis of the nonsyndromic form of the disease.

In a previous report, Jorgez and colleagues identified a 2p15 deletion encompassing *OTX1* in 6 subjects with genitourinary defects [15]. Three of these individuals had cryptorchidism and their genomic deletions also included *EHBP1* and *WDPC*. Other genitourinary anomalies of the three patients with cryptorchidism were variable including absent prepuce, micropenis, discontinuous raphe, penile cyst, hypoplastic scrotum, kidney stones or small testes. The three patients also had other defects including developmental delay, vision problems and dysmorphic facial features. Structural variations were also identified in studies of subjects with nonobstructive azoospermia or congenital genitourinary tract masculinization disorders from the same research group [14, 35]. In the study of nonobstructive azoospermia, 4 patients with microduplications and 4 with microdeletions of *E2F1* were identified among 110 affected individuals, but not among 78 fertile controls [35]. Two of the 8 patients with CNVs had cryptorchidism. Two non-synonymous mutations of *E2F1* (Ala102Thr and Gly393Ser) were also identified in three other patients, and one synonymous mutation (Leu415Leu) was identified in a patient with microduplication of *E2F1*. The patient with the Ala102Thr variant also had cryptorchidism. In the congenital genitourinary tract masculinization disorders study [14], copy number gains on Xq28 encompassing *VAMP7* were found in 4 of 296 patients. Two of them had idiopathic cryptorchidism, and the other two had hypospadias. They also found 1 case of hypospadias with *VAMP7* copy number gain in 28 distinct primary cultures of genital skin fibroblasts. All of the above three studies used array comparative genomic hybridization, a technology that enables efficient screening for CNVs, to discover the genomic variants. Other studies from Europe have also reported the microdeletions (2p14-p15, 2p15-16.1) in boys with cryptorchidism [36, 37]. However, all of these patients presented with other features besides cryptorchidism, including intellectual disability, developmental delay and/or dysmorphic features. In our study, subjects were excluded if there was evidence for other genital anomalies and/or other clinical features in addition to undescended testes.

Only autosomal CNVs were called and analyzed in our study, which may have led us to miss associated CNVs on the X or Y chromosome. The significant genes in our meta-analysis with CNVs that also passed QC in both groups and signal intensity visualization examination are *NUCB2* and *UPF2*, located at chromosome 11 and chromosome 10, respectively. However, these deletions were not validated by QuantStudio® 3D Digital PCR System with TaqMan CNV assays in our study samples, despite the signal intensity plots suggesting the presence of heterozygous deletions. The confidence score range of detected cases for these deletions is 12 to 55 (Table 2). The score numbers are lower in Group 2 cases with most of them less than 20. A confidence score of 10 has been suggested as a threshold to classify reliable CNV calls while the higher scores are more reliable and more likely to be replicated [38]. Most of our scores were less than the median score of 27.7 that was reported for deletions that could be replicated in the study of Ku et al. [38]. Due to different platforms with low overlapping SNP coverage that were used in genotyping Group 1 and Group 2 samples, we performed association tests separately in the two groups. Consequently, the whole study power was reduced compared to what it would have been if all samples had been genotyped on the same platform and some CNVs associated with disease may have been missed, even though we used meta-analysis to combine the two data sets. The use of SNP genotyping array data for CNV analysis is a common and acceptable approach [39–42], but the global CNV coverage of our SNP arrays varies. Cooper GM et al. [43] reported approximately 40 % and 80 % CNV coverage for Illumina chips of HumanHap550 and Human 1 M. Besides HumanHap550, the other chips we used, Human610-Quad and Human OminiExpress, have fewer SNPs compared to the Human 1 M, and therefore likely have less than 80 % global CNV coverage. Cooper GM et al. also reported that only two-thirds of detected CNVs by SNP data from Human 1 M could be validated in independent experiments [43], indicating that using SNP array data for CNV analysis may result in false positives, as may be the case in the present analysis.

## Conclusions

A sample size (800 cases and 2688 controls) greater than that of any other CNV analysis of nonsyndromic cryptorchidism failed to identify any associated variants, but weak effects at multiple genomic loci may still contribute to the etiology of this disease. It is also possible that CNVs are present but were not detected due to insufficient coverage by the SNP arrays we used and/or, the present analysis was underpowered to identify rare, strong effect CNVs that contribute to disease risk. Whole genome or exome sequencing, and comparative genomic hybridization are alternative approaches for discovery of disease-associated SNPs and CNVs, but beyond the scope of the present studies. It is possible that structural variation is more commonly associated with syndromic cryptorchidism, but our inability to validate the candidate CNVs in this analysis suggests that these variants are not a major cause of nonsyndromic cryptorchidism.

## Additional files

Additional file 1: genome-wide significant genes/loci in gene-based association tests in each group. Listed all the genes/loci with genome-wide significance (*p* < 5x10^−4^) in gene-based association tests in each group and their *p*-values. (DOCX 55 kb)
Additional file 2: genome-wide signficant genes/loci in meta-analysis. Listed all the genes/loci with genome-wide significant p-values (*p* < 5x10^−4^) in meta-analyses of gene-based association results, and their z-scores, *p*-values and the direction of effect in the two groups. (DOCX 57 kb)
Additional file 3: Array plot of Log R ration and B allele frequency for *TARP* and *TONSL*/*TONSL-AS1*. Showed the signal intensity plots of 2 CNVs in *TARP* and 1 CNV in *TONSL*/*TONSL-AS1*. The array plots did not pass visualization examination due to LRR close to 0, BAF cluster near 0.5, or both. (DOCX 141 kb)
Additional file 4: CNVs validation using TaqMan CNV assays and QuantStudio® 3D Digital PCR System. Listed the samples tested for CNV validating and the results. (XLSX 10 kb)
Additional file 5: CNV calls for Group 1 cases passed sample QC. Each column in Additional file 5 represents CNV location, SNPs numbers contained within the CNV, the length of the CNV, copy number (cn) of the CNV call, sample id, the starting marker identifier and the ending marker identifier in the CNV, and confidence score in PennCNV calling. (XLSX 653 kb)
Additional file 6: CNV calls for Group 1 controls passed sample QC. Each column in Additional file 6 represents CNV location, SNPs numbers contained within the CNV, the length of the CNV, copy number (cn) of the CNV call, sample id, the starting marker identifier and the ending marker identifier in the CNV, and confidence score in PennCNV calling. (XLSX 1544 kb)
Additional file 7: CNV calls for Group 2 cases passed sample QC. Each column in Additional file 7 represents CNV location, SNPs numbers contained within the CNV, the length of the CNV, copy number (cn) of the CNV call, sample id, the starting marker identifier and the ending marker identifier in the CNV, and confidence score in PennCNV calling. (XLSX 534 kb)
Additional file 8: CNV calls for Group 2 controls passed sample QC. Each column in Additional file 8 represents CNV location, SNPs numbers contained within the CNV, the length of the CNV, copy number (cn) of the CNV call, sample id, the starting marker identifier and the ending marker identifier in the CNV, and confidence score in PennCNV calling. (XLSX 1952 kb)

## Abbreviations

BAF: B allele frequency
CHOP: The Children’s Hospital of Philadelphia
CNV: Copy number variation
DGV: Database of genomic variants
GCWF: Guanine-cytosine base pair wave factor
GWAS: Genome-wide association study
HGDP: Human genome diversity project
LRR: Log R ratio
MDS: Multidimensional scaling
*NUCB2*: Nucleobindin 2
QC: Quality control
SD: Standard deviation
SDLRR: Standard deviation of log R ratio
SNP: Single nucleotide polymorphism
UPF2: UPF2 regulator of nonsense transcripts homolog (yeast).
