# Supplementary material for: Genomic copy number variation association study in Caucasian patients with nonsyndromic cryptorchidism
Source: BMC Urol. 2016 Oct 21;16:62. doi: 10.1186/s12894-016-0180-4 (PMC5073740; doi:10.1186/s12894-016-0180-4)
Supplement: Additional file 1: — genome-wide significant genes/loci in gene-based association tests in each group. Listed all the genes/loci with genome-wide significance (p < 5x10^−4^) in gene-based association tests in each group and their p-values. (DOCX 55 kb) [file 12894_2016_180_MOESM1_ESM.docx]

**Additional File 1: genome-wide significant genes/loci in gene-based association tests in each group**

**Group 1 Group 2**

| **Deletion** | | | | | **Duplication** | | **Deletion** | | | **Duplication** | | |
| --- | --- | --- | --- | --- | --- | --- | --- | --- | --- | --- | --- | --- |
| Gene | P value | | Gene | | | P value | | Gene | P value | | Gene | P value |
| UBR1 | 1.70E-22 | | SSTR4 | | | 1.43E-14 | | DPYSL4 | 6.36E-19 | | LINC00226 | 1.12E-97 |
| KBTBD10 | 1.96E-11 | | IRX1 | | | 2.77E-09 | | RAD52 | 7.39E-17 | | BC011773 | 1.34E-97 |
| BBS5 | 5.36E-11 | | ADAMTS1 | | | 8.31E-09 | | TRNA_Gln | 1.86E-16 | | Z49973 | 1.34E-97 |
| PICALM | 7.78E-10 | | LOC100506660 | | | 1.73E-08 | | TRNA_His | 1.32E-14 | | LINC00221 | 2.11E-96 |
| TTBK2 | 9.66E-10 | | SNORA75 | | | 1.73E-08 | | STK32C | 9.23E-13 | | abParts | 1.57E-85 |
| PARP8 | 1.31E-08 | | DDX11 | | | 4.25E-08 | | TRNA_Pseudo | 3.09E-12 | | ACAP3 | 7.71E-49 |
| BC043173 | 5.56E-08 | | MATN4 | | | 4.72E-08 | | PTK6 | 6.36E-11 | | CPSF3L | 3.01E-45 |
| CCDC104 | 1.25E-07 | | RBPJL | | | 4.72E-08 | | TET1 | 9.94E-11 | | PUSL1 | 3.40E-45 |
| TMEM65 | 1.44E-07 | | C1QTNF8 | | | 1.24E-07 | | SLC25A16 | 4.70E-10 | | MEGF6 | 2.85E-44 |
| USP34 | 2.99E-07 | | TRPM6 | | | 1.24E-07 | | TAOK1 | 4.70E-10 | | C2orf27A | 1.03E-42 |
| PPP1R9A | 7.94E-07 | | UPF3A | | | 1.94E-07 | | AQR | 2.59E-09 | | DVL1 | 4.89E-42 |
| STXBP5 | 1.12E-06 | | PCNT | | | 1.94E-07 | | SRMS | 2.59E-09 | | TAS1R3 | 4.89E-42 |
| U4atac | 1.84E-06 | | CHAMP1 | | | 7.22E-07 | | ASL | 1.04E-08 | | MXRA8 | 1.73E-40 |
| ADK | 4.09E-06 | | LINC00313 | | | 7.98E-07 | | SYNE1 | 2.49E-08 | | BRF1 | 1.85E-37 |
| BC045784 | 5.45E-06 | | LINC00319 | | | 7.98E-07 | | CCAR1 | 4.90E-08 | | GLTPD1 | 7.99E-37 |
| WNK1 | 6.14E-06 | | HCN2 | | | 3.05E-06 | | MICU1 | 4.90E-08 | | SCNN1D | 1.12E-35 |
| NUCB2 | 4.15E-05 | | DIP2A | | | 3.27E-06 | | MIR1256 | 4.90E-08 | | FOLH1B | 2.73E-35 |
| CYP7B1 | 5.35E-05 | | AX747730 | | | 3.27E-06 | | PTPRN2 | 1.09E-07 | | ANKRD20A9P | 7.00E-34 |
| TMEM33 | 0.00014509 | | CBS | | | 3.27E-06 | | ZNF430 | 1.09E-07 | | GKAP1 | 1.25E-29 |
| ZNF506 | 0.00014509 | | DNMT3A | | | 3.27E-06 | | MAPK8IP3 | 1.18E-07 | | MAPK8IP3 | 1.63E-24 |
| ACAP2 | 0.000164887 | | LINC00478 | | | 3.27E-06 | | CRCP | 2.29E-07 | | MRPS34 | 1.63E-24 |
| ATRNL1 | 0.000199609 | | LINC00317 | | | 3.27E-06 | | SNORD98 | 2.29E-07 | | NME3 | 1.63E-24 |
| NSRP1 | 0.000263497 | | PRMT2 | | | 3.27E-06 | | PTK7 | 4.73E-07 | | EME2 | 1.65E-24 |
| FLJ43860 | 0.000274913 | | PDE9A | | | 3.27E-06 | | DNA2 | 1.07E-06 | | DQ587539 | 2.78E-24 |
| SLC30A9 | 0.000383426 | | WDR4 | | | 3.27E-06 | | NEMF | 1.07E-06 | | NECAB2 | 2.97E-24 |
|  | |  | | PKNOX1 | | 3.27E-06 | | RUFY2 | 1.07E-06 | | DQ579288 | 1.25E-23 |
|  | |  | | U2AF1 | | 3.27E-06 | | TRNA_Asn | 1.07E-06 | | CDT1 | 9.74E-23 |
|  | |  | | NDUFV3 | | 3.27E-06 | | TCR-alphaV33.1 | 1.45E-06 | | APRT | 1.73E-22 |
|  | |  | | CRYAA | | 3.27E-06 | | KIF21B | 2.03E-06 | | ARHGEF16 | 5.47E-22 |
|  | |  | | C21orf105 | | 3.27E-06 | | TARP | 2.41E-06 | | SPSB3 | 5.47E-22 |
|  | |  | | DQ579288 | | 3.27E-06 | | TCRGC2 | 2.41E-06 | | MTMR3 | 1.11E-21 |
|  | |  | | BC035642 | | 3.27E-06 | | TRGC2 | 2.41E-06 | | SRMS | 1.68E-20 |
|  | |  | | BC024173 | | 3.27E-06 | | STXBP5L | 2.47E-06 | | PLEC | 5.09E-20 |
|  | |  | | BC048201 | | 3.27E-06 | | BAZ1A | 4.98E-06 | | PTK6 | 2.71E-19 |
|  | |  | | CACNA1H | | 6.83E-06 | | av27s1 | 6.87E-06 | | GALNS | 2.59E-18 |
|  | |  | | Y_RNA | | 7.21E-06 | | UPF2 | 8.64E-06 | | UBE2J2 | 4.13E-17 |
|  | |  | | KRTAP10-8 | | 1.34E-05 | | SLC19A1 | 9.83E-06 | | WDR90 | 1.94E-16 |
|  | |  | | ERG | | 1.34E-05 | | TCRGV | 2.08E-05 | | AGAP11 | 2.75E-16 |
|  | |  | | DQ598454 | | 1.34E-05 | | ATAD5 | 2.31E-05 | | C16orf13 | 5.99E-16 |
|  | |  | | HMGN1 | | 1.34E-05 | | C4orf34 | 2.31E-05 | | PRIC285 | 5.99E-16 |
|  | |  | | KRTAP10-4 | | 1.34E-05 | | FAM186A | 2.31E-05 | | RHOT2 | 9.78E-16 |
|  | |  | | C21orf89 | | 1.34E-05 | | LARP4 | 2.31E-05 | | PPDPF | 1.02E-15 |
|  | |  | | BC031638 | | 1.34E-05 | | LINC00200 | 2.31E-05 | | AK301549 | 1.67E-15 |
|  | |  | | LOC642852 | | 1.34E-05 | | PIGL | 2.31E-05 | | FAM195A | 1.67E-15 |
|  | |  | | MIR99A | | 1.34E-05 | | POLE2 | 2.31E-05 | | IGFALS | 8.32E-15 |
|  | |  | | KRTAP19-1 | | 1.34E-05 | | PSMA6 | 2.31E-05 | | NUBP2 | 8.32E-15 |
|  | |  | | LINC00323 | | 1.34E-05 | | TMEM116 | 2.31E-05 | | RHBDL1 | 8.32E-15 |
|  | |  | | DSCR6 | | 1.34E-05 | | AK096766 | 3.60E-05 | | JMJD8 | 8.43E-15 |
|  | |  | | pp9284 | | 1.34E-05 | | SEC24A | 3.63E-05 | | STUB1 | 8.43E-15 |
|  | |  | | SON | | 1.34E-05 | | AK298596 | 5.24E-05 | | WDR24 | 1.43E-14 |
|  | |  | | COL18A1 | | 1.34E-05 | | TONSL | 5.24E-05 | | EGFL7 | 6.98E-14 |
|  | |  | | PWP2 | | 1.34E-05 | | AK056558 | 0.000107 | | RAB40C | 1.16E-13 |
|  | |  | | B3GALT5 | | 1.34E-05 | | ALDH9A1 | 0.000107 | | AK128777 | 1.19E-13 |
|  | |  | | KRTAP24-1 | | 1.34E-05 | | AXDND1 | 0.000107 | | WFIKKN1 | 1.19E-13 |
|  | |  | | LOC100505746 | | 1.34E-05 | | BC040333 | 0.000107 | | AGPAT2 | 1.95E-13 |
|  | |  | | BACE2 | | 1.34E-05 | | CCDC88A | 0.000107 | | MIR126 | 1.95E-13 |
|  | |  | | LINC00162 | | 1.34E-05 | | DDX43 | 0.000107 | | TRNA_Gly | 1.96E-13 |
|  | |  | | C21orf59 | | 1.34E-05 | | EAPP | 0.000107 | | AF161442 | 2.01E-13 |
|  | |  | | KRTAP19-6 | | 1.34E-05 | | LOC494150 | 0.000107 | | TRNA | 4.07E-13 |
|  | |  | | PIGP | | 1.34E-05 | | MB21D1 | 0.000107 | | LOC100131320 | 5.39E-13 |
|  | |  | | DQ599834 | | 1.34E-05 | | MIR1288 | 0.000107 | | BSG | 1.01E-12 |
|  | |  | | LCA5L | | 1.34E-05 | | SRP54 | 0.000107 | | HLA-DRB1 | 1.26E-12 |
|  | |  | | PFKL | | 1.34E-05 | | TATDN3 | 0.000107 | | HLA-DRB5 | 1.26E-12 |
|  | |  | | GRIK1-AS1 | | 1.34E-05 | | TEFM | 0.000107 | | ZFP112 | 3.62E-12 |
|  | |  | | KCNJ6 | | 1.34E-05 | | TFCP2 | 0.000107 | | KCNQ2 | 4.63E-12 |
|  | |  | | ITSN1 | | 1.34E-05 | | TRPM7 | 0.000107 | | FAM132A | 1.23E-11 |
|  | |  | | C21orf49 | | 1.34E-05 | | UGDH | 0.000107 | | B3GALT6 | 2.06E-11 |
|  | |  | | TTC3 | | 1.34E-05 | | HLA-G | 0.000125 | | SDF4 | 2.06E-11 |
|  | |  | | PCBP3 | | 1.34E-05 | | HLA-H | 0.000125 | | LOC338579 | 8.36E-11 |
|  | |  | | DSCR10 | | 1.34E-05 | | HLA-J | 0.000125 | | ABCA2 | 9.64E-11 |
|  | |  | | FAM3B | | 1.34E-05 | | BMPR2 | 0.000147 | | C20orf195 | 1.59E-10 |
|  | |  | | KRTAP6-2 | | 1.34E-05 | | C20orf195 | 0.000147 | | HSD3BP4 | 2.38E-10 |
|  | |  | | DKFZp586E1322 | | 1.34E-05 | | FLVCR1-AS1 | 0.000147 | | RAB11FIP5 | 2.63E-10 |
|  | |  | | KRTAP26-1 | | 1.34E-05 | | CYP20A1 | 0.00015 | | TPSD1 | 2.63E-10 |
|  | |  | | IFNAR1 | | 1.34E-05 | | DPY19L4 | 0.00015 | | NHLRC4 | 2.69E-10 |
|  | |  | | GCFC1 | | 1.34E-05 | | PDS5A | 0.00015 | | PIGQ | 2.69E-10 |
|  | |  | | TRPM2 | | 1.34E-05 | | AK097625 | 0.000194 | | STEAP1B | 4.00E-10 |
|  | |  | | S100B | | 1.34E-05 | | USP34 | 0.000436 | | ZNF285 | 4.00E-10 |
|  | |  | | OLIG1 | | 1.34E-05 | | AL831889 | 0.000495 | | C9orf139 | 7.90E-10 |
|  | |  | | GPX4 | | 1.34E-05 | | BAZ1B | 0.000495 | | FUT7 | 7.90E-10 |
|  | |  | | POFUT2 | | 1.34E-05 | | BX537811 | 0.000495 | | RXRA | 2.11E-09 |
|  | |  | | KRTAP22-2 | | 1.34E-05 | | EYA3 | 0.000495 | | HAGHL | 3.25E-09 |
|  | |  | | SLC19A1 | | 1.34E-05 | | HNRNPH3 | 0.000495 | | NARFL | 3.25E-09 |
|  | |  | | DQ577420 | | 1.34E-05 | | IDE | 0.000495 | | CCDC78 | 5.63E-09 |
|  | |  | | SLC5A3 | | 1.34E-05 | | ITFG1 | 0.000495 | | CLIC3 | 9.20E-09 |
|  | |  | | SLC37A1 | | 1.34E-05 | | KLB | 0.000495 | | KLF16 | 1.46E-08 |
|  | |  | | IL10RB | | 1.34E-05 | | LIAS | 0.000495 | | CADM2 | 2.45E-08 |
|  | |  | | C21orf2 | | 1.34E-05 | | LOC401127 | 0.000495 | | MAMDC4 | 2.45E-08 |
|  | |  | | HUNK | | 1.34E-05 | | NOP58 | 0.000495 | | PHPT1 | 2.45E-08 |
|  | |  | | LOC100288432 | | 1.34E-05 | | NUP210L | 0.000495 | | HCN2 | 3.98E-08 |
|  | |  | | KRTAP13-2 | | 1.34E-05 | | PAFAH1B1 | 0.000495 | | REXO1 | 4.00E-08 |
|  | |  | | HLCS | | 1.34E-05 | | PPM1D | 0.000495 | | EDF1 | 6.53E-08 |
|  | |  | | PTTG1IP | | 1.34E-05 | | SLC30A6 | 0.000495 | | FAM173A | 6.56E-08 |
|  | |  | | ADARB1 | | 1.34E-05 | | SNORD11 | 0.000495 | | ANKRD30B | 9.52E-08 |
|  | |  | | KRTAP10-5 | | 1.34E-05 | | SNORD11B | 0.000495 | | AL360260 | 1.07E-07 |
|  | |  | | FAM207A | | 1.34E-05 | | SNORD70 | 0.000495 | | BTBD6 | 1.08E-07 |
|  | |  | | KRTAP10-12 | | 1.34E-05 | | SNX6 | 0.000495 | | FBXL16 | 1.74E-07 |
|  | |  | | DSCAM-AS1 | | 1.34E-05 | | SPAST | 0.000495 | | EMB | 2.04E-07 |
|  | |  | | KRTAP10-9 | | 1.34E-05 | | USP37 | 0.000495 | | KIAA0528 | 2.84E-07 |
|  | |  | | BC039377 | | 1.34E-05 | | WBSCR27 | 0.000495 | | METRN | 3.11E-07 |
|  | |  | | RRP1 | | 1.34E-05 | | WBSCR28 | 0.000495 | | SYT1 | 5.01E-07 |
|  | |  | | SOD1 | | 1.34E-05 | | ZFP14 | 0.000495 | | GPSM1 | 7.56E-07 |
|  | |  | | AL109792 | | 1.34E-05 | | ZNF143 | 0.000495 | | C9orf142 | 8.08E-07 |
|  | |  | | KRTAP20-1 | | 1.34E-05 | | ZNF501 | 0.000495 | | GRM5 | 1.23E-06 |
|  | |  | | TMPRSS3 | | 1.34E-05 | | ZNF714 | 0.000495 | | AK293020 | 1.30E-06 |
|  | |  | | CLDN14 | | 1.34E-05 | | ZNF85 | 0.000495 | | HLA-DRB6 | 1.30E-06 |
|  | |  | | KRTAP12-4 | | 1.34E-05 | |  |  | | C9orf172 | 2.02E-06 |
|  | |  | | DM119543 | | 1.34E-05 | |  |  | | LCN10 | 2.02E-06 |
|  | |  | | Z49981 | | 1.34E-05 | |  |  | | LCN8 | 2.02E-06 |
|  | |  | | PSMG1 | | 1.34E-05 | |  |  | | LCN6 | 2.10E-06 |
|  | |  | | AL355711 | | 1.34E-05 | |  |  | | LOC100128593 | 2.10E-06 |
|  | |  | | KRTAP19-5 | | 1.34E-05 | |  |  | | PSPC1 | 2.19E-06 |
|  | |  | | KRTAP27-1 | | 1.34E-05 | |  |  | | EEF1D | 3.29E-06 |
|  | |  | | BRWD1-IT2 | | 1.34E-05 | |  |  | | ZNF441 | 4.54E-06 |
|  | |  | | TMEM50B | | 1.34E-05 | |  |  | | GPR123 | 5.10E-06 |
|  | |  | | ATP5J | | 1.34E-05 | |  |  | | C16orf11 | 5.48E-06 |
|  | |  | | LINC00307 | | 1.34E-05 | |  |  | | DNLZ | 5.48E-06 |
|  | |  | | LOC284837 | | 1.34E-05 | |  |  | | NAPRT1 | 5.48E-06 |
|  | |  | | MIR155 | | 1.34E-05 | |  |  | | BC034020 | 8.76E-06 |
|  | |  | | BC033260 | | 1.34E-05 | |  |  | | TIGD5 | 8.76E-06 |
|  | |  | | KRTAP6-1 | | 1.34E-05 | |  |  | | GPC5 | 8.81E-06 |
|  | |  | | C21orf33 | | 1.34E-05 | |  |  | | CARD9 | 8.85E-06 |
|  | |  | | AX746823 | | 1.34E-05 | |  |  | | TPRN | 8.85E-06 |
|  | |  | | TSPEAR | | 1.34E-05 | |  |  | | TUBGCP2 | 1.43E-05 |
|  | |  | | DSCR9 | | 1.34E-05 | |  |  | | C9orf86 | 1.43E-05 |
|  | |  | | MX1 | | 1.34E-05 | |  |  | | EXD3 | 1.43E-05 |
|  | |  | | LINC00479 | | 1.34E-05 | |  |  | | FAM69B | 1.43E-05 |
|  | |  | | SIM2 | | 1.34E-05 | |  |  | | NPDC1 | 1.43E-05 |
|  | |  | | CSTB | | 1.34E-05 | |  |  | | NUDT14 | 1.43E-05 |
|  | |  | | MRPS6 | | 1.34E-05 | |  |  | | SOLH | 1.43E-05 |
|  | |  | | KRTAP15-1 | | 1.34E-05 | |  |  | | LCN15 | 2.31E-05 |
|  | |  | | SH3BGR | | 1.34E-05 | |  |  | | ADAM8 | 2.34E-05 |
|  | |  | | KRTAP12-3 | | 1.34E-05 | |  |  | | CD151 | 2.34E-05 |
|  | |  | | C21orf90 | | 1.34E-05 | |  |  | | CHTF18 | 3.73E-05 |
|  | |  | | KRTAP19-8 | | 1.34E-05 | |  |  | | LCN12 | 3.73E-05 |
|  | |  | | KRTAP22-1 | | 1.34E-05 | |  |  | | POLR2L | 3.73E-05 |
|  | |  | | IFNAR2 | | 1.34E-05 | |  |  | | RPUSD1 | 3.73E-05 |
|  | |  | | AX748362 | | 1.34E-05 | |  |  | | OR4S2 | 3.80E-05 |
|  | |  | | AX813477 | | 1.34E-05 | |  |  | | BC064596 | 6.03E-05 |
|  | |  | | POLR2E | | 1.34E-05 | |  |  | | C9orf140 | 6.56E-05 |
|  | |  | | WRB | | 1.34E-05 | |  |  | | ENTPD2 | 6.56E-05 |
|  | |  | | TFF1 | | 1.34E-05 | |  |  | | INTS1 | 6.56E-05 |
|  | |  | | KRTAP13-1 | | 1.34E-05 | |  |  | | MSLNL | 6.56E-05 |
|  | |  | | SCAF4 | | 1.34E-05 | |  |  | | AK127339 | 9.75E-05 |
|  | |  | | OLIG2 | | 1.34E-05 | |  |  | | C1QTNF9B | 1.00E-04 |
|  | |  | | AK027145 | | 1.34E-05 | |  |  | | C1QTNF9B-AS1 | 1.00E-04 |
|  | |  | | KRTAP21-3 | | 1.34E-05 | |  |  | | LOC649395 | 1.00E-04 |
|  | |  | | BRWD1 | | 1.34E-05 | |  |  | | MIPEP | 1.00E-04 |
|  | |  | | C21orf128 | | 1.34E-05 | |  |  | | C19orf26 | 0.000105 |
|  | |  | | AK057604 | | 1.34E-05 | |  |  | | EFCAB4A | 0.000105 |
|  | |  | | DONSON | | 1.34E-05 | |  |  | | JA429539 | 0.000105 |
|  | |  | | LINC00308 | | 1.34E-05 | |  |  | | MICALL2 | 0.000105 |
|  | |  | | DSCR8 | | 1.34E-05 | |  |  | | STK11 | 0.000105 |
|  | |  | | KRTAP19-4 | | 1.34E-05 | |  |  | | UAP1L1 | 0.000105 |
|  | |  | | CRYZL1 | | 1.34E-05 | |  |  | | OR4C11 | 0.000154 |
|  | |  | | ABCG1 | | 1.34E-05 | |  |  | | OR4P4 | 0.000154 |
|  | |  | | LINC00160 | | 1.34E-05 | |  |  | | CACNA1H | 0.000158 |
|  | |  | | MIS18A | | 1.34E-05 | |  |  | | LCNL1 | 0.000168 |
|  | |  | | MIR125B2 | | 1.34E-05 | |  |  | | LRP1B | 0.000168 |
|  | |  | | CHODL-AS1 | | 1.34E-05 | |  |  | | MIR3176 | 0.000168 |
|  | |  | | KRTAP11-1 | | 1.34E-05 | |  |  | | PTGDS | 0.000168 |
|  | |  | | C21orf88 | | 1.34E-05 | |  |  | | ATP5D | 0.000255 |
|  | |  | | LINC00515 | | 1.34E-05 | |  |  | | ANAPC2 | 0.000268 |
|  | |  | | C21orf119 | | 1.34E-05 | |  |  | | CHID1 | 0.000268 |
|  | |  | | KRTAP20-2 | | 1.34E-05 | |  |  | | GMEB2 | 0.000268 |
|  | |  | | AGPAT3 | | 1.34E-05 | |  |  | | MIR202 | 0.000268 |
|  | |  | | MIRLET7C | | 1.34E-05 | |  |  | | SIGIRR | 0.000268 |
|  | |  | | PDXK | | 1.34E-05 | |  |  | | SNHG7 | 0.000268 |
|  | |  | | NRIP1 | | 1.34E-05 | |  |  | | SNORA17 | 0.000268 |
|  | |  | | ETS2 | | 1.34E-05 | |  |  | | SNORA43 | 0.000268 |
|  | |  | | KRTAP10-2 | | 1.34E-05 | |  |  | | SSNA1 | 0.000268 |
|  | |  | | KRTAP10-1 | | 1.34E-05 | |  |  | | TNFRSF18 | 0.000268 |
|  | |  | | DQ590668 | | 1.34E-05 | |  |  | | GALM | 0.000334 |
|  | |  | | MORC3 | | 1.34E-05 | |  |  | | LOC100288123 | 0.000413 |
|  | |  | | COL6A2 | | 1.34E-05 | |  |  | | PYCRL | 0.000413 |
|  | |  | | KRTAP19-7 | | 1.34E-05 | |  |  | | AURKAIP1 | 0.000429 |
|  | |  | | HSF2BP | | 1.34E-05 | |  |  | | MIDN | 0.000429 |
|  | |  | | DQ601137 | | 1.34E-05 | |  |  | |  |  |
|  | |  | | RSPH1 | | 1.34E-05 | |  |  | |  |  |
|  | |  | | C21orf63 | | 1.34E-05 | |  |  | |  |  |
|  | |  | | KRTAP12-2 | | 1.34E-05 | |  |  | |  |  |
|  | |  | | SUMO3 | | 1.34E-05 | |  |  | |  |  |
|  | |  | | KRTAP13-4 | | 1.34E-05 | |  |  | |  |  |
|  | |  | | KRTAP10-6 | | 1.34E-05 | |  |  | |  |  |
|  | |  | | IFNGR2 | | 1.34E-05 | |  |  | |  |  |
|  | |  | | KRTAP6-3 | | 1.34E-05 | |  |  | |  |  |
|  | |  | | CLCN7 | | 1.34E-05 | |  |  | |  |  |
|  | |  | | TFF2 | | 1.34E-05 | |  |  | |  |  |
|  | |  | | LOC100129027 | | 1.34E-05 | |  |  | |  |  |
|  | |  | | MIR4327 | | 1.34E-05 | |  |  | |  |  |
|  | |  | | LOC100506385 | | 1.34E-05 | |  |  | |  |  |
|  | |  | | CLDN17 | | 1.34E-05 | |  |  | |  |  |
|  | |  | | SSR4P1 | | 1.34E-05 | |  |  | |  |  |
|  | |  | | ICOSLG | | 1.34E-05 | |  |  | |  |  |
|  | |  | | BC058002 | | 1.34E-05 | |  |  | |  |  |
|  | |  | | RRP1B | | 1.34E-05 | |  |  | |  |  |
|  | |  | | GART | | 1.34E-05 | |  |  | |  |  |
|  | |  | | LINC00158 | | 1.34E-05 | |  |  | |  |  |
|  | |  | | KRTAP25-1 | | 1.34E-05 | |  |  | |  |  |
|  | |  | | IGSF5 | | 1.34E-05 | |  |  | |  |  |
|  | |  | | TRAPPC10 | | 1.34E-05 | |  |  | |  |  |
|  | |  | | CHODL | | 1.34E-05 | |  |  | |  |  |
|  | |  | | KRTAP21-2 | | 1.34E-05 | |  |  | |  |  |
|  | |  | | C21orf91-OT1 | | 1.34E-05 | |  |  | |  |  |
|  | |  | | GABPA | | 1.34E-05 | |  |  | |  |  |
|  | |  | | TMPRSS2 | | 1.34E-05 | |  |  | |  |  |
|  | |  | | KRTAP10-11 | | 1.34E-05 | |  |  | |  |  |
|  | |  | | TMPRSS15 | | 1.34E-05 | |  |  | |  |  |
|  | |  | | MX2 | | 1.34E-05 | |  |  | |  |  |
|  | |  | | Z49979 | | 1.34E-05 | |  |  | |  |  |
|  | |  | | C21orf15 | | 1.34E-05 | |  |  | |  |  |
|  | |  | | UBASH3A | | 1.34E-05 | |  |  | |  |  |
|  | |  | | UBE2G2 | | 1.34E-05 | |  |  | |  |  |
|  | |  | | C21orf54 | | 1.34E-05 | |  |  | |  |  |
|  | |  | | LINC00163 | | 1.34E-05 | |  |  | |  |  |
|  | |  | | LINC00111 | | 1.34E-05 | |  |  | |  |  |
|  | |  | | CCDC154 | | 1.34E-05 | |  |  | |  |  |
|  | |  | | KRTAP12-1 | | 1.34E-05 | |  |  | |  |  |
|  | |  | | DQ588725 | | 1.34E-05 | |  |  | |  |  |
|  | |  | | HSPA13 | | 1.34E-05 | |  |  | |  |  |
|  | |  | | DNMT3L | | 1.34E-05 | |  |  | |  |  |
|  | |  | | TCP10L | | 1.34E-05 | |  |  | |  |  |
|  | |  | | ATP5O | | 1.34E-05 | |  |  | |  |  |
|  | |  | | KRTAP19-2 | | 1.34E-05 | |  |  | |  |  |
|  | |  | | GRIK1 | | 1.34E-05 | |  |  | |  |  |
|  | |  | | GRIK1-AS2 | | 1.34E-05 | |  |  | |  |  |
|  | |  | | YBEY | | 1.34E-05 | |  |  | |  |  |
|  | |  | | BC041455 | | 1.34E-05 | |  |  | |  |  |
|  | |  | | GCFC1-AS1 | | 1.34E-05 | |  |  | |  |  |
|  | |  | | MRPL39 | | 1.34E-05 | |  |  | |  |  |
|  | |  | | KRTAP10-7 | | 1.34E-05 | |  |  | |  |  |
|  | |  | | RBM11 | | 1.34E-05 | |  |  | |  |  |
|  | |  | | KRTAP20-3 | | 1.34E-05 | |  |  | |  |  |
|  | |  | | MIR4760 | | 1.34E-05 | |  |  | |  |  |
|  | |  | | AK124194 | | 1.34E-05 | |  |  | |  |  |
|  | |  | | KRTAP10-3 | | 1.34E-05 | |  |  | |  |  |
|  | |  | | MIR3197 | | 1.34E-05 | |  |  | |  |  |
|  | |  | | C21orf62 | | 1.34E-05 | |  |  | |  |  |
|  | |  | | JAM2 | | 1.34E-05 | |  |  | |  |  |
|  | |  | | DNAJC28 | | 1.34E-05 | |  |  | |  |  |
|  | |  | | COL18A1-AS1 | | 1.34E-05 | |  |  | |  |  |
|  | |  | | MIR1301 | | 1.34E-05 | |  |  | |  |  |
|  | |  | | MIR155HG | | 1.34E-05 | |  |  | |  |  |
|  | |  | | C21orf91 | | 1.34E-05 | |  |  | |  |  |
|  | |  | | KRTAP23-1 | | 1.34E-05 | |  |  | |  |  |
|  | |  | | KRTAP20-4 | | 1.34E-05 | |  |  | |  |  |
|  | |  | | C2CD2 | | 1.34E-05 | |  |  | |  |  |
|  | |  | | intersectin1longform | | 1.34E-05 | |  |  | |  |  |
|  | |  | | DSCR3 | | 1.34E-05 | |  |  | |  |  |
|  | |  | | ZNF295 | | 1.34E-05 | |  |  | |  |  |
|  | |  | | KRTAP8-1 | | 1.34E-05 | |  |  | |  |  |
|  | |  | | DSCR4 | | 1.34E-05 | |  |  | |  |  |
|  | |  | | KRTAP13-3 | | 1.34E-05 | |  |  | |  |  |
|  | |  | | LRRC3 | | 1.34E-05 | |  |  | |  |  |
|  | |  | | BC041449 | | 1.34E-05 | |  |  | |  |  |
|  | |  | | RUNX1-IT1 | | 1.34E-05 | |  |  | |  |  |
|  | |  | | DYRK1A | | 1.34E-05 | |  |  | |  |  |
|  | |  | | SYNJ1 | | 1.34E-05 | |  |  | |  |  |
|  | |  | | CHAF1B | | 1.34E-05 | |  |  | |  |  |
|  | |  | | PCP4 | | 1.34E-05 | |  |  | |  |  |
|  | |  | | KRTAP10-10 | | 1.34E-05 | |  |  | |  |  |
|  | |  | | SNORA80 | | 1.34E-05 | |  |  | |  |  |
|  | |  | | CLDN8 | | 1.34E-05 | |  |  | |  |  |
|  | |  | | ZNF295-AS1 | | 1.34E-05 | |  |  | |  |  |
|  | |  | | SNORD74 | | 1.34E-05 | |  |  | |  |  |
|  | |  | | SNORA62 | | 1.34E-05 | |  |  | |  |  |
|  | |  | | COL6A1 | | 1.34E-05 | |  |  | |  |  |
|  | |  | | PLAC4 | | 1.34E-05 | |  |  | |  |  |
|  | |  | | KRTAP21-1 | | 1.34E-05 | |  |  | |  |  |
|  | |  | | FTCD | | 1.34E-05 | |  |  | |  |  |
|  | |  | | KCNJ15 | | 1.34E-05 | |  |  | |  |  |
|  | |  | | TFF3 | | 1.34E-05 | |  |  | |  |  |
|  | |  | | LOC100506334 | | 1.34E-05 | |  |  | |  |  |
|  | |  | | C21orf58 | | 1.34E-05 | |  |  | |  |  |
|  | |  | | KRTAP19-3 | | 1.34E-05 | |  |  | |  |  |
|  | |  | | CLIC6 | | 1.34E-05 | |  |  | |  |  |
|  | |  | | C21orf67 | | 1.34E-05 | |  |  | |  |  |
|  | |  | | LINC00112 | | 1.34E-05 | |  |  | |  |  |
|  | |  | | LINC00310 | | 1.34E-05 | |  |  | |  |  |
|  | |  | | GPR144 | | 1.91E-05 | |  |  | |  |  |
|  | |  | | NR5A1 | | 1.91E-05 | |  |  | |  |  |
|  | |  | | LONP1 | | 2.52E-05 | |  |  | |  |  |
|  | |  | | BC043580 | | 2.55E-05 | |  |  | |  |  |
|  | |  | | DQ579969 | | 2.55E-05 | |  |  | |  |  |
|  | |  | | TRNA_Sup | | 2.55E-05 | |  |  | |  |  |
|  | |  | | UMODL1 | | 2.55E-05 | |  |  | |  |  |
|  | |  | | USP25 | | 2.55E-05 | |  |  | |  |  |
|  | |  | | KLC3 | | 2.75E-05 | |  |  | |  |  |
|  | |  | | C21orf56 | | 5.48E-05 | |  |  | |  |  |
|  | |  | | SBNO2 | | 5.48E-05 | |  |  | |  |  |
|  | |  | | CDH15 | | 5.48E-05 | |  |  | |  |  |
|  | |  | | UNKL | | 5.48E-05 | |  |  | |  |  |
|  | |  | | HMHA1 | | 5.48E-05 | |  |  | |  |  |
|  | |  | | LINC00304 | | 5.48E-05 | |  |  | |  |  |
|  | |  | | MCM3AP-AS1 | | 5.48E-05 | |  |  | |  |  |
|  | |  | | ADSSL1 | | 5.48E-05 | |  |  | |  |  |
|  | |  | | LSS | | 5.48E-05 | |  |  | |  |  |
|  | |  | | ZNF876P | | 5.48E-05 | |  |  | |  |  |
|  | |  | | C16orf91 | | 5.48E-05 | |  |  | |  |  |
|  | |  | | ACSF3 | | 5.48E-05 | |  |  | |  |  |
|  | |  | | MCM3AP | | 5.48E-05 | |  |  | |  |  |
|  | |  | | ADAMTS5 | | 9.44E-05 | |  |  | |  |  |
|  | |  | | AIRE | | 9.44E-05 | |  |  | |  |  |
|  | |  | | AK074469 | | 9.44E-05 | |  |  | |  |  |
|  | |  | | APP | | 9.44E-05 | |  |  | |  |  |
|  | |  | | AX747935 | | 9.44E-05 | |  |  | |  |  |
|  | |  | | BACH1 | | 9.44E-05 | |  |  | |  |  |
|  | |  | | BC014150 | | 9.44E-05 | |  |  | |  |  |
|  | |  | | BC034802 | | 9.44E-05 | |  |  | |  |  |
|  | |  | | BC047600 | | 9.44E-05 | |  |  | |  |  |
|  | |  | | BC101420 | | 9.44E-05 | |  |  | |  |  |
|  | |  | | BTG3 | | 9.44E-05 | |  |  | |  |  |
|  | |  | | C21orf37 | | 9.44E-05 | |  |  | |  |  |
|  | |  | | C21orf7 | | 9.44E-05 | |  |  | |  |  |
|  | |  | | CBR1 | | 9.44E-05 | |  |  | |  |  |
|  | |  | | CCT8 | | 9.44E-05 | |  |  | |  |  |
|  | |  | | CXADR | | 9.44E-05 | |  |  | |  |  |
|  | |  | | CYYR1 | | 9.44E-05 | |  |  | |  |  |
|  | |  | | D21S2088E | | 9.44E-05 | |  |  | |  |  |
|  | |  | | DSCAM | | 9.44E-05 | |  |  | |  |  |
|  | |  | | GLTSCR1 | | 9.44E-05 | |  |  | |  |  |
|  | |  | | INF2 | | 9.44E-05 | |  |  | |  |  |
|  | |  | | ITGB2 | | 9.44E-05 | |  |  | |  |  |
|  | |  | | KRTAP7-1 | | 9.44E-05 | |  |  | |  |  |
|  | |  | | LINC00113 | | 9.44E-05 | |  |  | |  |  |
|  | |  | | LINC00159 | | 9.44E-05 | |  |  | |  |  |
|  | |  | | LINC00161 | | 9.44E-05 | |  |  | |  |  |
|  | |  | | LINC00189 | | 9.44E-05 | |  |  | |  |  |
|  | |  | | LINC00314 | | 9.44E-05 | |  |  | |  |  |
|  | |  | | LINC00320 | | 9.44E-05 | |  |  | |  |  |
|  | |  | | LIPI | | 9.44E-05 | |  |  | |  |  |
|  | |  | | LTN1 | | 9.44E-05 | |  |  | |  |  |
|  | |  | | MIR4759 | | 9.44E-05 | |  |  | |  |  |
|  | |  | | MIR802 | | 9.44E-05 | |  |  | |  |  |
|  | |  | | MRAP | | 9.44E-05 | |  |  | |  |  |
|  | |  | | N6AMT1 | | 9.44E-05 | |  |  | |  |  |
|  | |  | | NCAM2 | | 9.44E-05 | |  |  | |  |  |
|  | |  | | PRDM15 | | 9.44E-05 | |  |  | |  |  |
|  | |  | | RIPK4 | | 9.44E-05 | |  |  | |  |  |
|  | |  | | RUNX1 | | 9.44E-05 | |  |  | |  |  |
|  | |  | | RWDD2B | | 9.44E-05 | |  |  | |  |  |
|  | |  | | SAMSN1 | | 9.44E-05 | |  |  | |  |  |
|  | |  | | SETD4 | | 9.44E-05 | |  |  | |  |  |
|  | |  | | TIAM1 | | 9.44E-05 | |  |  | |  |  |
|  | |  | | URB1 | | 9.44E-05 | |  |  | |  |  |
|  | |  | | USP16 | | 9.44E-05 | |  |  | |  |  |
|  | |  | | 5S_rRNA | | 9.70E-05 | |  |  | |  |  |
|  | |  | | POLRMT | | 9.70E-05 | |  |  | |  |  |
|  | |  | | BC084558 | | 0.000108 | |  |  | |  |  |
|  | |  | | DOPEY2 | | 0.000108 | |  |  | |  |  |
|  | |  | | LOC146336 | | 0.000108 | |  |  | |  |  |
|  | |  | | SSTR5 | | 0.000108 | |  |  | |  |  |
|  | |  | | ABCC13 | | 0.00011 | |  |  | |  |  |
|  | |  | | DQ587539 | | 0.00011 | |  |  | |  |  |
|  | |  | | MKNK2 | | 0.000223 | |  |  | |  |  |
|  | |  | | IQSEC3 | | 0.000223 | |  |  | |  |  |
|  | |  | | LOC90925 | | 0.000223 | |  |  | |  |  |
|  | |  | | LOC100133669 | | 0.000223 | |  |  | |  |  |
|  | |  | | LY6E | | 0.000223 | |  |  | |  |  |
|  | |  | | DQ591735 | | 0.000242 | |  |  | |  |  |
|  | |  | | MIR3156-3 | | 0.000242 | |  |  | |  |  |
|  | |  | | POTED | | 0.000242 | |  |  | |  |  |
|  | |  | | CSMD1 | | 0.000295 | |  |  | |  |  |
|  | |  | | U6 | | 0.000312 | |  |  | |  |  |
|  | |  | | CBR3 | | 0.000343 | |  |  | |  |  |
|  | |  | | LINC00114 | | 0.000343 | |  |  | |  |  |
|  | |  | | LOC100133286 | | 0.000343 | |  |  | |  |  |
|  | |  | | LOC100506428 | | 0.000343 | |  |  | |  |  |
|  | |  | | PLEC | | 0.000344 | |  |  | |  |  |
|  | |  | | Mir_562 | | 0.00037 | |  |  | |  |  |
|  | |  | | Metazoa_SRP | | 0.000426 | |  |  | |  |  |
