# Supplementary material for: Genomic copy number variation association study in Caucasian patients with nonsyndromic cryptorchidism
Source: BMC Urol. 2016 Oct 21;16:62. doi: 10.1186/s12894-016-0180-4 (PMC5073740; doi:10.1186/s12894-016-0180-4)
Supplement: Additional file 2: — genome-wide signficant genes/loci in meta-analysis. Listed all the genes/loci with genome-wide significant p-values (p < 5x10^−4^) in meta-analyses of gene-based association results, and their z-scores, p-values and the direction of effect in the two groups. (DOCX 57 kb) [file 12894_2016_180_MOESM2_ESM.docx]

**Additional File 2: genome-wide significant genes/loci in meta-analysis**

|  |  | **Deletion** |  |  |  | **Duplication** |  |  |
| --- | --- | --- | --- | --- | --- | --- | --- | --- |
|  |  |  |  |  |  |  |  |  |
| **Gene** | **Zscore** | **P-value** | **Direction** |  | **Gene** | **Zscore** | **P-value** | **Direction** |
| TRNA_His | -6.524 | 6.83E-11 | -- |  | LINC00226^*^ | -12.676 | 8.01E-37 | +- |
| RAD52 | 6.222 | 4.92E-10 | ++ |  | BC011773^*^ | -12.671 | 8.60E-37 | +- |
| USP34 | 6.191 | 5.99E-10 | ++ |  | Z49973^*^ | -12.671 | 8.60E-37 | +- |
| TRNA_Pseudo | -6.056 | 1.39E-09 | -- |  | abParts^*^ | -12.427 | 1.88E-35 | +- |
| KBTBD10 | 5.955 | 2.60E-09 | ++ |  | LINC00221^*^ | -11.61 | 3.65E-31 | +- |
| BBS5 | 5.841 | 5.20E-09 | ++ |  | DQ579288 | 10.027 | 1.16E-23 | ++ |
| TET1 | 5.59 | 2.27E-08 | ++ |  | DQ587539 | 9.524 | 1.67E-21 | ++ |
| TTBK2 | 5.497 | 3.86E-08 | ++ |  | FOLH1B | 8.896 | 5.78E-19 | ++ |
| UPF2 | 5.109 | 3.25E-07 | ++ |  | ADAMTS1 | 5.195 | 2.05E-07 | ++ |
| SLC25A16 | 4.877 | 1.08E-06 | ++ |  | WDR4 | 4.835 | 1.33E-06 | ++ |
| AQR | 4.697 | 2.65E-06 | ++ |  | PCNT | 4.769 | 1.85E-06 | ++ |
| ATAD5 | 4.603 | 4.17E-06 | ++ |  | LINC00313 | 4.564 | 5.01E-06 | ++ |
| NUCB2 | 4.503 | 6.71E-06 | ++ |  | LINC00319 | 4.564 | 5.01E-06 | ++ |
| SYNE1 | 4.46 | 8.21E-06 | ++ |  | LOC728323 | 4.438 | 9.07E-06 | ++ |
| TARP | 4.459 | 8.24E-06 | ++ |  | PRMT2 | 4.348 | 1.37E-05 | ++ |
| TCRGC2 | 4.459 | 8.24E-06 | ++ |  | AX747730 | 4.348 | 1.37E-05 | ++ |
| TRGC2 | 4.459 | 8.24E-06 | ++ |  | BC035642 | 4.348 | 1.37E-05 | ++ |
| SRP54 | 4.44 | 8.99E-06 | ++ |  | C21orf105 | 4.348 | 1.37E-05 | ++ |
| CCAR1 | 4.384 | 1.17E-05 | ++ |  | CBS | 4.348 | 1.37E-05 | ++ |
| TEFM | 4.374 | 1.22E-05 | ++ |  | CRYAA | 4.348 | 1.37E-05 | ++ |
| SNORD98 | 4.205 | 2.61E-05 | ++ |  | DIP2A | 4.348 | 1.37E-05 | ++ |
| MICU1 | 4.169 | 3.06E-05 | ++ |  | LINC00317 | 4.348 | 1.37E-05 | ++ |
| MIR1256 | 4.169 | 3.06E-05 | ++ |  | NDUFV3 | 4.348 | 1.37E-05 | ++ |
| MAPK8IP3 | 4.106 | 4.02E-05 | ++ |  | PDE9A | 4.348 | 1.37E-05 | ++ |
| NSRP1 | 4.073 | 4.65E-05 | ++ |  | PKNOX1 | 4.348 | 1.37E-05 | ++ |
| DNA2 | 4.017 | 5.90E-05 | ++ |  | U2AF1 | 4.348 | 1.37E-05 | ++ |
| RUFY2 | 4.017 | 5.90E-05 | ++ |  | GALM | -4.23 | 2.33E-05 | -- |
| hADV29S1 | -3.96 | 7.50E-05 | -- |  | OR4S2 | -4.18 | 2.91E-05 | -- |
| SLC19A1 | 3.946 | 7.95E-05 | ++ |  | HSPA13 | 4.12 | 3.79E-05 | ++ |
| hADV36S1 | -3.915 | 9.04E-05 | -- |  | SLC37A1 | 4.12 | 3.79E-05 | ++ |
| AV4S1 | -3.914 | 9.07E-05 | -- |  | CLDN17 | 4.12 | 3.79E-05 | ++ |
| TCRGV | 3.908 | 9.31E-05 | ++ |  | pp9284 | 4.12 | 3.79E-05 | ++ |
| hADV38S2 | -3.892 | 9.95E-05 | -- |  | TMPRSS2 | 4.12 | 3.79E-05 | ++ |
| ADK | 3.87 | 0.000109 | ++ |  | BC058002 | 4.12 | 3.79E-05 | ++ |
| DL491467 | 3.859 | 0.0001138 | ++ |  | CHAF1B | 4.12 | 3.79E-05 | ++ |
| DQ590492 | 3.859 | 0.0001138 | ++ |  | CLDN14 | 4.12 | 3.79E-05 | ++ |
| EEF1A1 | 3.859 | 0.0001138 | ++ |  | HLCS | 4.12 | 3.79E-05 | ++ |
| AK291611 | 3.74 | 0.0001838 | ++ |  | MORC3 | 4.12 | 3.79E-05 | ++ |
| X06774 | 3.74 | 0.0001838 | ++ |  | SIM2 | 4.12 | 3.79E-05 | ++ |
| ZNF506 | 3.712 | 0.000206 | ++ |  | ADARB1 | 4.12 | 3.79E-05 | ++ |
| AK298596 | 3.706 | 0.0002103 | ++ |  | AK124194 | 4.12 | 3.79E-05 | ++ |
| TONSL | 3.706 | 0.0002103 | ++ |  | BC031638 | 4.12 | 3.79E-05 | ++ |
| LARP4 | 3.606 | 0.0003115 | ++ |  | BRWD1 | 4.12 | 3.79E-05 | ++ |
| PIGL | 3.606 | 0.0003115 | ++ |  | C21orf58 | 4.12 | 3.79E-05 | ++ |
| C4orf34 | 3.606 | 0.0003115 | ++ |  | C21orf62 | 4.12 | 3.79E-05 | ++ |
| DOK6 | 3.594 | 0.0003255 | ++ |  | C21orf67 | 4.12 | 3.79E-05 | ++ |
| AK096766 | 3.544 | 0.0003942 | ++ |  | C21orf89 | 4.12 | 3.79E-05 | ++ |
| CDK19 | 3.538 | 0.0004039 | ++ |  | COL18A1-AS1 | 4.12 | 3.79E-05 | ++ |
| BAZ1A | 3.53 | 0.0004149 | ++ |  | COL6A1 | 4.12 | 3.79E-05 | ++ |
|  |  |  |  |  | COL6A2 | 4.12 | 3.79E-05 | ++ |
|  |  |  |  |  | DKFZp586E1322 | 4.12 | 3.79E-05 | ++ |
|  |  |  |  |  | FAM207A | 4.12 | 3.79E-05 | ++ |
|  |  |  |  |  | FTCD | 4.12 | 3.79E-05 | ++ |
|  |  |  |  |  | GRIK1 | 4.12 | 3.79E-05 | ++ |
|  |  |  |  |  | GRIK1-AS1 | 4.12 | 3.79E-05 | ++ |
|  |  |  |  |  | GRIK1-AS2 | 4.12 | 3.79E-05 | ++ |
|  |  |  |  |  | LINC00162 | 4.12 | 3.79E-05 | ++ |
|  |  |  |  |  | LINC00163 | 4.12 | 3.79E-05 | ++ |
|  |  |  |  |  | LOC100129027 | 4.12 | 3.79E-05 | ++ |
|  |  |  |  |  | LOC100505746 | 4.12 | 3.79E-05 | ++ |
|  |  |  |  |  | LOC642852 | 4.12 | 3.79E-05 | ++ |
|  |  |  |  |  | NRIP1 | 4.12 | 3.79E-05 | ++ |
|  |  |  |  |  | OLIG2 | 4.12 | 3.79E-05 | ++ |
|  |  |  |  |  | PCBP3 | 4.12 | 3.79E-05 | ++ |
|  |  |  |  |  | POFUT2 | 4.12 | 3.79E-05 | ++ |
|  |  |  |  |  | SSR4P1 | 4.12 | 3.79E-05 | ++ |
|  |  |  |  |  | YBEY | 4.12 | 3.79E-05 | ++ |
|  |  |  |  |  | Z49979 | 4.12 | 3.79E-05 | ++ |
|  |  |  |  |  | AX748362 | 4.12 | 3.79E-05 | ++ |
|  |  |  |  |  | C21orf128 | 4.12 | 3.79E-05 | ++ |
|  |  |  |  |  | C2CD2 | 4.12 | 3.79E-05 | ++ |
|  |  |  |  |  | MIS18A | 4.12 | 3.79E-05 | ++ |
|  |  |  |  |  | ZNF295 | 4.12 | 3.79E-05 | ++ |
|  |  |  |  |  | ZNF295-AS1 | 4.12 | 3.79E-05 | ++ |
|  |  |  |  |  | ABCG1 | 4.12 | 3.79E-05 | ++ |
|  |  |  |  |  | AGPAT3 | 4.12 | 3.79E-05 | ++ |
|  |  |  |  |  | AK027145 | 4.12 | 3.79E-05 | ++ |
|  |  |  |  |  | AK057604 | 4.12 | 3.79E-05 | ++ |
|  |  |  |  |  | AL109792 | 4.12 | 3.79E-05 | ++ |
|  |  |  |  |  | AL355711 | 4.12 | 3.79E-05 | ++ |
|  |  |  |  |  | ATP5J | 4.12 | 3.79E-05 | ++ |
|  |  |  |  |  | ATP5O | 4.12 | 3.79E-05 | ++ |
|  |  |  |  |  | AX746823 | 4.12 | 3.79E-05 | ++ |
|  |  |  |  |  | AX813477 | 4.12 | 3.79E-05 | ++ |
|  |  |  |  |  | B3GALT5 | 4.12 | 3.79E-05 | ++ |
|  |  |  |  |  | BACE2 | 4.12 | 3.79E-05 | ++ |
|  |  |  |  |  | BC033260 | 4.12 | 3.79E-05 | ++ |
|  |  |  |  |  | BC039377 | 4.12 | 3.79E-05 | ++ |
|  |  |  |  |  | BC041449 | 4.12 | 3.79E-05 | ++ |
|  |  |  |  |  | BC041455 | 4.12 | 3.79E-05 | ++ |
|  |  |  |  |  | BRWD1-IT2 | 4.12 | 3.79E-05 | ++ |
|  |  |  |  |  | C21orf119 | 4.12 | 3.79E-05 | ++ |
|  |  |  |  |  | C21orf2 | 4.12 | 3.79E-05 | ++ |
|  |  |  |  |  | C21orf33 | 4.12 | 3.79E-05 | ++ |
|  |  |  |  |  | C21orf49 | 4.12 | 3.79E-05 | ++ |
|  |  |  |  |  | C21orf54 | 4.12 | 3.79E-05 | ++ |
|  |  |  |  |  | C21orf59 | 4.12 | 3.79E-05 | ++ |
|  |  |  |  |  | C21orf63 | 4.12 | 3.79E-05 | ++ |
|  |  |  |  |  | C21orf88 | 4.12 | 3.79E-05 | ++ |
|  |  |  |  |  | C21orf90 | 4.12 | 3.79E-05 | ++ |
|  |  |  |  |  | C21orf91 | 4.12 | 3.79E-05 | ++ |
|  |  |  |  |  | C21orf91-OT1 | 4.12 | 3.79E-05 | ++ |
|  |  |  |  |  | CHODL | 4.12 | 3.79E-05 | ++ |
|  |  |  |  |  | CHODL-AS1 | 4.12 | 3.79E-05 | ++ |
|  |  |  |  |  | CLDN8 | 4.12 | 3.79E-05 | ++ |
|  |  |  |  |  | CLIC6 | 4.12 | 3.79E-05 | ++ |
|  |  |  |  |  | CRYZL1 | 4.12 | 3.79E-05 | ++ |
|  |  |  |  |  | CSTB | 4.12 | 3.79E-05 | ++ |
|  |  |  |  |  | DM119543 | 4.12 | 3.79E-05 | ++ |
|  |  |  |  |  | DNAJC28 | 4.12 | 3.79E-05 | ++ |
|  |  |  |  |  | DNMT3L | 4.12 | 3.79E-05 | ++ |
|  |  |  |  |  | DONSON | 4.12 | 3.79E-05 | ++ |
|  |  |  |  |  | DQ577420 | 4.12 | 3.79E-05 | ++ |
|  |  |  |  |  | DQ588725 | 4.12 | 3.79E-05 | ++ |
|  |  |  |  |  | DQ590668 | 4.12 | 3.79E-05 | ++ |
|  |  |  |  |  | DQ598454 | 4.12 | 3.79E-05 | ++ |
|  |  |  |  |  | DQ599834 | 4.12 | 3.79E-05 | ++ |
|  |  |  |  |  | DQ601137 | 4.12 | 3.79E-05 | ++ |
|  |  |  |  |  | DSCAM-AS1 | 4.12 | 3.79E-05 | ++ |
|  |  |  |  |  | DSCR10 | 4.12 | 3.79E-05 | ++ |
|  |  |  |  |  | DSCR3 | 4.12 | 3.79E-05 | ++ |
|  |  |  |  |  | DSCR4 | 4.12 | 3.79E-05 | ++ |
|  |  |  |  |  | DSCR6 | 4.12 | 3.79E-05 | ++ |
|  |  |  |  |  | DSCR8 | 4.12 | 3.79E-05 | ++ |
|  |  |  |  |  | DSCR9 | 4.12 | 3.79E-05 | ++ |
|  |  |  |  |  | DYRK1A | 4.12 | 3.79E-05 | ++ |
|  |  |  |  |  | ERG | 4.12 | 3.79E-05 | ++ |
|  |  |  |  |  | ETS2 | 4.12 | 3.79E-05 | ++ |
|  |  |  |  |  | FAM3B | 4.12 | 3.79E-05 | ++ |
|  |  |  |  |  | GABPA | 4.12 | 3.79E-05 | ++ |
|  |  |  |  |  | GART | 4.12 | 3.79E-05 | ++ |
|  |  |  |  |  | GCFC1 | 4.12 | 3.79E-05 | ++ |
|  |  |  |  |  | GCFC1-AS1 | 4.12 | 3.79E-05 | ++ |
|  |  |  |  |  | HMGN1 | 4.12 | 3.79E-05 | ++ |
|  |  |  |  |  | HSF2BP | 4.12 | 3.79E-05 | ++ |
|  |  |  |  |  | HUNK | 4.12 | 3.79E-05 | ++ |
|  |  |  |  |  | ICOSLG | 4.12 | 3.79E-05 | ++ |
|  |  |  |  |  | IFNAR1 | 4.12 | 3.79E-05 | ++ |
|  |  |  |  |  | IFNAR2 | 4.12 | 3.79E-05 | ++ |
|  |  |  |  |  | IGSF5 | 4.12 | 3.79E-05 | ++ |
|  |  |  |  |  | IL10RB | 4.12 | 3.79E-05 | ++ |
|  |  |  |  |  | intersectin1longform | 4.12 | 3.79E-05 | ++ |
|  |  |  |  |  | ITSN1 | 4.12 | 3.79E-05 | ++ |
|  |  |  |  |  | JAM2 | 4.12 | 3.79E-05 | ++ |
|  |  |  |  |  | KCNJ15 | 4.12 | 3.79E-05 | ++ |
|  |  |  |  |  | KCNJ6 | 4.12 | 3.79E-05 | ++ |
|  |  |  |  |  | KRTAP10-1 | 4.12 | 3.79E-05 | ++ |
|  |  |  |  |  | KRTAP10-10 | 4.12 | 3.79E-05 | ++ |
|  |  |  |  |  | KRTAP10-11 | 4.12 | 3.79E-05 | ++ |
|  |  |  |  |  | KRTAP10-12 | 4.12 | 3.79E-05 | ++ |
|  |  |  |  |  | KRTAP10-2 | 4.12 | 3.79E-05 | ++ |
|  |  |  |  |  | KRTAP10-3 | 4.12 | 3.79E-05 | ++ |
|  |  |  |  |  | KRTAP10-4 | 4.12 | 3.79E-05 | ++ |
|  |  |  |  |  | KRTAP10-5 | 4.12 | 3.79E-05 | ++ |
|  |  |  |  |  | KRTAP10-6 | 4.12 | 3.79E-05 | ++ |
|  |  |  |  |  | KRTAP10-7 | 4.12 | 3.79E-05 | ++ |
|  |  |  |  |  | KRTAP10-8 | 4.12 | 3.79E-05 | ++ |
|  |  |  |  |  | KRTAP10-9 | 4.12 | 3.79E-05 | ++ |
|  |  |  |  |  | KRTAP11-1 | 4.12 | 3.79E-05 | ++ |
|  |  |  |  |  | KRTAP12-1 | 4.12 | 3.79E-05 | ++ |
|  |  |  |  |  | KRTAP12-2 | 4.12 | 3.79E-05 | ++ |
|  |  |  |  |  | KRTAP12-3 | 4.12 | 3.79E-05 | ++ |
|  |  |  |  |  | KRTAP12-4 | 4.12 | 3.79E-05 | ++ |
|  |  |  |  |  | KRTAP13-1 | 4.12 | 3.79E-05 | ++ |
|  |  |  |  |  | KRTAP13-2 | 4.12 | 3.79E-05 | ++ |
|  |  |  |  |  | KRTAP13-3 | 4.12 | 3.79E-05 | ++ |
|  |  |  |  |  | KRTAP13-4 | 4.12 | 3.79E-05 | ++ |
|  |  |  |  |  | KRTAP15-1 | 4.12 | 3.79E-05 | ++ |
|  |  |  |  |  | KRTAP19-1 | 4.12 | 3.79E-05 | ++ |
|  |  |  |  |  | KRTAP19-2 | 4.12 | 3.79E-05 | ++ |
|  |  |  |  |  | KRTAP19-3 | 4.12 | 3.79E-05 | ++ |
|  |  |  |  |  | KRTAP19-4 | 4.12 | 3.79E-05 | ++ |
|  |  |  |  |  | KRTAP19-5 | 4.12 | 3.79E-05 | ++ |
|  |  |  |  |  | KRTAP19-6 | 4.12 | 3.79E-05 | ++ |
|  |  |  |  |  | KRTAP19-7 | 4.12 | 3.79E-05 | ++ |
|  |  |  |  |  | KRTAP19-8 | 4.12 | 3.79E-05 | ++ |
|  |  |  |  |  | KRTAP20-1 | 4.12 | 3.79E-05 | ++ |
|  |  |  |  |  | KRTAP20-2 | 4.12 | 3.79E-05 | ++ |
|  |  |  |  |  | KRTAP20-3 | 4.12 | 3.79E-05 | ++ |
|  |  |  |  |  | KRTAP20-4 | 4.12 | 3.79E-05 | ++ |
|  |  |  |  |  | KRTAP21-1 | 4.12 | 3.79E-05 | ++ |
|  |  |  |  |  | KRTAP21-2 | 4.12 | 3.79E-05 | ++ |
|  |  |  |  |  | KRTAP21-3 | 4.12 | 3.79E-05 | ++ |
|  |  |  |  |  | KRTAP22-1 | 4.12 | 3.79E-05 | ++ |
|  |  |  |  |  | KRTAP22-2 | 4.12 | 3.79E-05 | ++ |
|  |  |  |  |  | KRTAP23-1 | 4.12 | 3.79E-05 | ++ |
|  |  |  |  |  | KRTAP24-1 | 4.12 | 3.79E-05 | ++ |
|  |  |  |  |  | KRTAP25-1 | 4.12 | 3.79E-05 | ++ |
|  |  |  |  |  | KRTAP26-1 | 4.12 | 3.79E-05 | ++ |
|  |  |  |  |  | KRTAP27-1 | 4.12 | 3.79E-05 | ++ |
|  |  |  |  |  | KRTAP6-1 | 4.12 | 3.79E-05 | ++ |
|  |  |  |  |  | KRTAP6-2 | 4.12 | 3.79E-05 | ++ |
|  |  |  |  |  | KRTAP6-3 | 4.12 | 3.79E-05 | ++ |
|  |  |  |  |  | KRTAP8-1 | 4.12 | 3.79E-05 | ++ |
|  |  |  |  |  | LCA5L | 4.12 | 3.79E-05 | ++ |
|  |  |  |  |  | LINC00111 | 4.12 | 3.79E-05 | ++ |
|  |  |  |  |  | LINC00112 | 4.12 | 3.79E-05 | ++ |
|  |  |  |  |  | LINC00158 | 4.12 | 3.79E-05 | ++ |
|  |  |  |  |  | LINC00160 | 4.12 | 3.79E-05 | ++ |
|  |  |  |  |  | LINC00307 | 4.12 | 3.79E-05 | ++ |
|  |  |  |  |  | LINC00308 | 4.12 | 3.79E-05 | ++ |
|  |  |  |  |  | LINC00310 | 4.12 | 3.79E-05 | ++ |
|  |  |  |  |  | LINC00323 | 4.12 | 3.79E-05 | ++ |
|  |  |  |  |  | LINC00479 | 4.12 | 3.79E-05 | ++ |
|  |  |  |  |  | LINC00515 | 4.12 | 3.79E-05 | ++ |
|  |  |  |  |  | LOC100288432 | 4.12 | 3.79E-05 | ++ |
|  |  |  |  |  | LOC100506334 | 4.12 | 3.79E-05 | ++ |
|  |  |  |  |  | LOC100506385 | 4.12 | 3.79E-05 | ++ |
|  |  |  |  |  | LOC284837 | 4.12 | 3.79E-05 | ++ |
|  |  |  |  |  | LRRC3 | 4.12 | 3.79E-05 | ++ |
|  |  |  |  |  | MIR125B2 | 4.12 | 3.79E-05 | ++ |
|  |  |  |  |  | MIR155 | 4.12 | 3.79E-05 | ++ |
|  |  |  |  |  | MIR155HG | 4.12 | 3.79E-05 | ++ |
|  |  |  |  |  | MIR3197 | 4.12 | 3.79E-05 | ++ |
|  |  |  |  |  | MIR4327 | 4.12 | 3.79E-05 | ++ |
|  |  |  |  |  | MIR4760 | 4.12 | 3.79E-05 | ++ |
|  |  |  |  |  | MIR99A | 4.12 | 3.79E-05 | ++ |
|  |  |  |  |  | MIRLET7C | 4.12 | 3.79E-05 | ++ |
|  |  |  |  |  | MRPL39 | 4.12 | 3.79E-05 | ++ |
|  |  |  |  |  | MRPS6 | 4.12 | 3.79E-05 | ++ |
|  |  |  |  |  | MX1 | 4.12 | 3.79E-05 | ++ |
|  |  |  |  |  | MX2 | 4.12 | 3.79E-05 | ++ |
|  |  |  |  |  | OLIG1 | 4.12 | 3.79E-05 | ++ |
|  |  |  |  |  | PCP4 | 4.12 | 3.79E-05 | ++ |
|  |  |  |  |  | PDXK | 4.12 | 3.79E-05 | ++ |
|  |  |  |  |  | PFKL | 4.12 | 3.79E-05 | ++ |
|  |  |  |  |  | PIGP | 4.12 | 3.79E-05 | ++ |
|  |  |  |  |  | PLAC4 | 4.12 | 3.79E-05 | ++ |
|  |  |  |  |  | PTTG1IP | 4.12 | 3.79E-05 | ++ |
|  |  |  |  |  | PWP2 | 4.12 | 3.79E-05 | ++ |
|  |  |  |  |  | RRP1 | 4.12 | 3.79E-05 | ++ |
|  |  |  |  |  | RRP1B | 4.12 | 3.79E-05 | ++ |
|  |  |  |  |  | RSPH1 | 4.12 | 3.79E-05 | ++ |
|  |  |  |  |  | RUNX1-IT1 | 4.12 | 3.79E-05 | ++ |
|  |  |  |  |  | S100B | 4.12 | 3.79E-05 | ++ |
|  |  |  |  |  | SCAF4 | 4.12 | 3.79E-05 | ++ |
|  |  |  |  |  | SH3BGR | 4.12 | 3.79E-05 | ++ |
|  |  |  |  |  | SLC5A3 | 4.12 | 3.79E-05 | ++ |
|  |  |  |  |  | SNORA62 | 4.12 | 3.79E-05 | ++ |
|  |  |  |  |  | SNORA80 | 4.12 | 3.79E-05 | ++ |
|  |  |  |  |  | SOD1 | 4.12 | 3.79E-05 | ++ |
|  |  |  |  |  | SON | 4.12 | 3.79E-05 | ++ |
|  |  |  |  |  | SUMO3 | 4.12 | 3.79E-05 | ++ |
|  |  |  |  |  | SYNJ1 | 4.12 | 3.79E-05 | ++ |
|  |  |  |  |  | TCP10L | 4.12 | 3.79E-05 | ++ |
|  |  |  |  |  | TFF1 | 4.12 | 3.79E-05 | ++ |
|  |  |  |  |  | TFF2 | 4.12 | 3.79E-05 | ++ |
|  |  |  |  |  | TFF3 | 4.12 | 3.79E-05 | ++ |
|  |  |  |  |  | TMPRSS15 | 4.12 | 3.79E-05 | ++ |
|  |  |  |  |  | TMPRSS3 | 4.12 | 3.79E-05 | ++ |
|  |  |  |  |  | TRAPPC10 | 4.12 | 3.79E-05 | ++ |
|  |  |  |  |  | TRPM2 | 4.12 | 3.79E-05 | ++ |
|  |  |  |  |  | TSPEAR | 4.12 | 3.79E-05 | ++ |
|  |  |  |  |  | TTC3 | 4.12 | 3.79E-05 | ++ |
|  |  |  |  |  | UBASH3A | 4.12 | 3.79E-05 | ++ |
|  |  |  |  |  | UBE2G2 | 4.12 | 3.79E-05 | ++ |
|  |  |  |  |  | WRB | 4.12 | 3.79E-05 | ++ |
|  |  |  |  |  | Z49981 | 4.12 | 3.79E-05 | ++ |
|  |  |  |  |  | MIR3156-3 | 4.106 | 4.03E-05 | ++ |
|  |  |  |  |  | DQ579969 | 4.01 | 6.06E-05 | ++ |
|  |  |  |  |  | BC043580 | 4.01 | 6.06E-05 | ++ |
|  |  |  |  |  | UMODL1 | 4.01 | 6.06E-05 | ++ |
|  |  |  |  |  | USP25 | 4.01 | 6.06E-05 | ++ |
|  |  |  |  |  | Mir_562 | 4.001 | 6.30E-05 | ++ |
|  |  |  |  |  | TRNA^*^ | -3.982 | 6.84E-05 | +- |
|  |  |  |  |  | BC024173 | 3.966 | 7.31E-05 | ++ |
|  |  |  |  |  | BC048201 | 3.966 | 7.31E-05 | ++ |
|  |  |  |  |  | RBM11 | 3.881 | 0.000104 | ++ |
|  |  |  |  |  | SNORD74 | 3.881 | 0.000104 | ++ |
|  |  |  |  |  | IFNGR2 | 3.881 | 0.000104 | ++ |
|  |  |  |  |  | PSMG1 | 3.881 | 0.000104 | ++ |
|  |  |  |  |  | TMEM50B | 3.881 | 0.000104 | ++ |
|  |  |  |  |  | C21orf56 | 3.876 | 0.000106 | ++ |
|  |  |  |  |  | LSS | 3.876 | 0.000106 | ++ |
|  |  |  |  |  | MCM3AP | 3.876 | 0.000106 | ++ |
|  |  |  |  |  | MCM3AP-AS1 | 3.876 | 0.000106 | ++ |
|  |  |  |  |  | 5S_rRNA | 3.845 | 0.0001204 | ++ |
|  |  |  |  |  | ANKRD20A11P | 3.824 | 0.0001312 | ++ |
|  |  |  |  |  | U3 | 3.782 | 0.0001556 | ++ |
|  |  |  |  |  | CBR1 | 3.777 | 0.0001584 | ++ |
|  |  |  |  |  | SETD4 | 3.777 | 0.0001584 | ++ |
|  |  |  |  |  | D21S2088E | 3.777 | 0.0001584 | ++ |
|  |  |  |  |  | MIR802 | 3.777 | 0.0001584 | ++ |
|  |  |  |  |  | LTN1 | 3.777 | 0.0001584 | ++ |
|  |  |  |  |  | BACH1 | 3.777 | 0.0001584 | ++ |
|  |  |  |  |  | C21orf7 | 3.777 | 0.0001584 | ++ |
|  |  |  |  |  | CCT8 | 3.777 | 0.0001584 | ++ |
|  |  |  |  |  | DSCAM | 3.777 | 0.0001584 | ++ |
|  |  |  |  |  | LINC00189 | 3.777 | 0.0001584 | ++ |
|  |  |  |  |  | RWDD2B | 3.777 | 0.0001584 | ++ |
|  |  |  |  |  | USP16 | 3.777 | 0.0001584 | ++ |
|  |  |  |  |  | AK074469 | 3.777 | 0.0001584 | ++ |
|  |  |  |  |  | APP | 3.777 | 0.0001584 | ++ |
|  |  |  |  |  | ITGB2 | 3.777 | 0.0001584 | ++ |
|  |  |  |  |  | TIAM1 | 3.777 | 0.0001584 | ++ |
|  |  |  |  |  | LINC00314 | 3.777 | 0.0001584 | ++ |
|  |  |  |  |  | ADAMTS5 | 3.777 | 0.0001584 | ++ |
|  |  |  |  |  | AX747935 | 3.777 | 0.0001584 | ++ |
|  |  |  |  |  | BC034802 | 3.777 | 0.0001584 | ++ |
|  |  |  |  |  | BC047600 | 3.777 | 0.0001584 | ++ |
|  |  |  |  |  | BC101420 | 3.777 | 0.0001584 | ++ |
|  |  |  |  |  | BTG3 | 3.777 | 0.0001584 | ++ |
|  |  |  |  |  | C21orf37 | 3.777 | 0.0001584 | ++ |
|  |  |  |  |  | CXADR | 3.777 | 0.0001584 | ++ |
|  |  |  |  |  | LINC00113 | 3.777 | 0.0001584 | ++ |
|  |  |  |  |  | LINC00159 | 3.777 | 0.0001584 | ++ |
|  |  |  |  |  | LINC00161 | 3.777 | 0.0001584 | ++ |
|  |  |  |  |  | MIR4759 | 3.777 | 0.0001584 | ++ |
|  |  |  |  |  | MRAP | 3.777 | 0.0001584 | ++ |
|  |  |  |  |  | N6AMT1 | 3.777 | 0.0001584 | ++ |
|  |  |  |  |  | PRDM15 | 3.777 | 0.0001584 | ++ |
|  |  |  |  |  | RIPK4 | 3.777 | 0.0001584 | ++ |
|  |  |  |  |  | URB1 | 3.777 | 0.0001584 | ++ |
|  |  |  |  |  | AIRE | 3.777 | 0.0001584 | ++ |
|  |  |  |  |  | BC014150 | 3.777 | 0.0001584 | ++ |
|  |  |  |  |  | CYYR1 | 3.777 | 0.0001584 | ++ |
|  |  |  |  |  | KRTAP7-1 | 3.777 | 0.0001584 | ++ |
|  |  |  |  |  | LINC00320 | 3.777 | 0.0001584 | ++ |
|  |  |  |  |  | DOPEY2 | 3.77 | 0.0001632 | ++ |
|  |  |  |  |  | ABCC13 | 3.75 | 0.0001771 | ++ |
|  |  |  |  |  | Y_RNA | 3.74 | 0.000184 | ++ |
|  |  |  |  |  | SLC19A1 | 3.738 | 0.0001858 | ++ |
|  |  |  |  |  | C21orf15 | 3.738 | 0.0001858 | ++ |
|  |  |  |  |  | DQ590589 | 3.646 | 0.000266 | ++ |
|  |  |  |  |  | TRNA_Sup | 3.628 | 0.0002855 | ++ |
|  |  |  |  |  | LINC00478^*^ | 3.55 | 0.0003851 | 0 |
|  |  |  |  |  | CBR3 | 3.548 | 0.0003888 | ++ |
|  |  |  |  |  | LOC100133286 | 3.548 | 0.0003888 | ++ |
|  |  |  |  |  | LOC100506428 | 3.548 | 0.0003888 | ++ |
|  |  |  |  |  | RUNX1 | 3.539 | 0.0004023 | ++ |
|  |  |  |  |  | SAMSN1 | 3.539 | 0.0004023 | ++ |

* Gene/locus with inconsistent effect direction or no effect direction information in group 1 and group 2.
