# Supplementary figures and images for: Genomic copy number variation association study in Caucasian patients with nonsyndromic cryptorchidism
Source: BMC Urol. 2016 Oct 21;16:62. doi: 10.1186/s12894-016-0180-4 (PMC5073740; doi:10.1186/s12894-016-0180-4)

**Additional File 3: Array plot of Log R ratio and B allele frequency for *TARP* and *TONSL*/*TONSL-AS1***


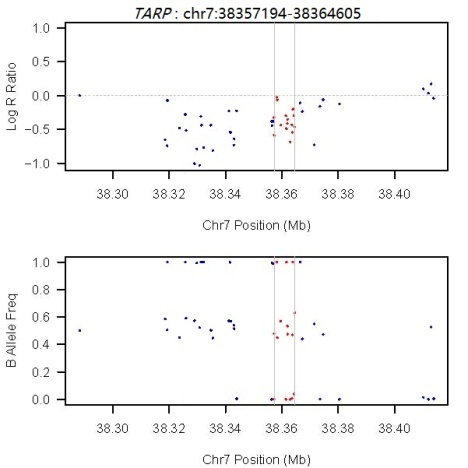

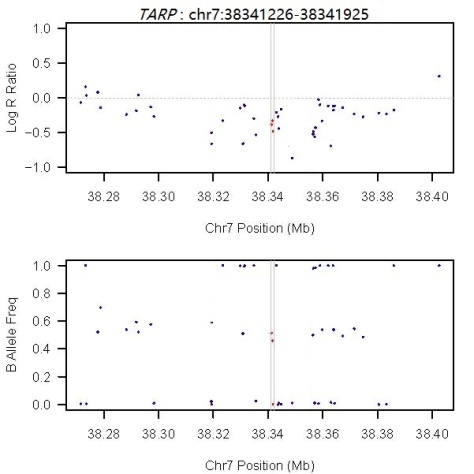

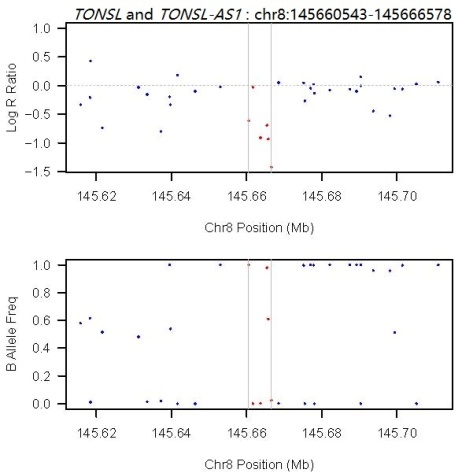

Supplement: Additional file 3: — Array plot of Log R ration and B allele frequency for TARP and TONSL/TONSL-AS1. Showed the signal intensity plots of 2 CNVs in TARP and 1 CNV in TONSL/TONSL-AS1. The array plots did not pass visualization examination due to LRR close to 0, BAF cluster near 0.5, or both. (DOCX 141 kb) [file 12894_2016_180_MOESM3_ESM.docx]
